# Natural Variation Identifies *ICARUS1*, a Universal Gene Required for Cell Proliferation and Growth at High Temperatures in *Arabidopsis thaliana*

**DOI:** 10.1371/journal.pgen.1005085

**Published:** 2015-05-07

**Authors:** Wangsheng Zhu, Israel Ausin, Andrei Seleznev, Belén Méndez-Vigo, F. Xavier Picó, Sridevi Sureshkumar, Vignesh Sundaramoorthi, Dieter Bulach, David Powell, Torsten Seemann, Carlos Alonso-Blanco, Sureshkumar Balasubramanian

**Affiliations:** 1 School of Biological Sciences, Monash University, Victoria, Australia; 2 Centro Nacional de Biotecnología (CNB), Consejo Superior de Investigaciones Científicas (CSIC), Madrid, Spain; 3 Estación Biológica de Doñana (EBD), Consejo Superior de Investigaciones Científicas (CSIC), Seville, Spain; 4 Victorian Bioinformatics Consortium, Monash University, Victoria, Australia; Harvard University, UNITED STATES

## Abstract

Plants are highly sensitive to environmental changes and even small variations in ambient temperature have severe consequences on their growth and development. Temperature affects multiple aspects of plant development, but the processes and mechanisms underlying thermo-sensitive growth responses are mostly unknown. Here we exploit natural variation in *Arabidopsis thaliana* to identify and characterize novel components and processes mediating thermo-sensitive growth responses in plants. Phenotypic screening of wild accessions identified several strains displaying pleiotropic growth defects, at cellular and organism levels, specifically at high ambient temperatures. Positional cloning and characterization of the underlying gene revealed that *ICARUS1* (*ICA1*), which encodes a protein of the tRNA^His^ guanylyl transferase (Thg1) superfamily, is required for plant growth at high temperatures. Transcriptome and gene marker analyses together with DNA content measurements show that *ICA1* loss-of-function results in down regulation of cell cycle associated genes at high temperatures, which is linked with a block in G2/M transition and endoreduplication. In addition, plants with mutations in *ICA1* show enhanced sensitivity to DNA damage. Characterization of additional strains that carry lesions in *ICA1*, but display normal growth, shows that alternative splicing is likely to alleviate the deleterious effects of some natural mutations. Furthermore, analyses of worldwide and regional collections of natural accessions indicate that *ICA1* loss-of-function has arisen several times independently, and that these occur at high frequency in some local populations. Overall our results suggest that *ICA1*-mediated-modulation of fundamental processes such as tRNA^His^ maturation, modify plant growth responses to temperature changes in a quantitative and reversible manner, in natural populations.

## Author Summary

The increase in average temperatures across the globe has been predicted to have negative impacts on agricultural productivity. Therefore, there is a need to understand the molecular mechanisms that underlie plant growth responses to varying temperature regimes. At present, very little is known about the genes and pathways that modulate thermo-sensory growth responses in plants. In this article, the authors exploit natural variation in the commonly occurring weed thale cress (*Arabidopsis thaliana)* and identify a gene referred to as *ICARUS1* to be required for plant growth at higher ambient temperatures. Plants carrying lesions in this gene stop growing at high temperatures and revert to growth when temperatures reduce. Using a combination of computational, molecular and cell biological approaches, the authors demonstrate that allelic variation at *ICARUS1*, which encodes an enzyme required for the fundamental biochemical process of tRNA^His^ maturation, underlies variation in thermo-sensory growth responses of *A*. *thaliana*. Furthermore, the authors discover that the deleterious impact of a natural mutation in *ICARUS1* is suppressed through alternative splicing, thus suggesting the potential for alternative splicing to buffer the impacts of some natural mutations. These results support that modulation of fundamental processes, in addition to transcriptional regulation, mediate thermo-sensory growth responses in plants.

## Introduction

Environmental perturbations can often reveal cryptic phenotypes, which in turn can uncover mechanisms associated with environmental regulation of growth and development [1–5]. Light and temperature are the two key environmental factors that have major impacts on plant development. The molecular mechanisms associated with light signaling and its regulation of plant development is very well studied [6–9]. In contrast, temperature response has been studied traditionally at extreme conditions characterized by heat shock response or cold stress response [10–13]. However, even small differences in ambient growth temperature can have profound effects on plant growth and development [12, 14, 15].

Vernalization, the acceleration of flowering in response to exposure to winter-like temperatures, is one of the developmental processes well studied at the molecular level [16, 17]. In contrast to this response to extreme temperatures, very little is known about the molecular mechanisms underlying thermo-sensory responses within moderate growth temperature ranges [14]. Plants grown at higher ambient temperatures display elongated hypocotyls and petioles, increased leaf serration, as well as early flowering [18–21]. Thermo-sensory responses have been suggested to involve chromatin remodeling involving histone dynamics [22–24]. For example, the incorporation and eviction of histone H2A.Z onto the nucleosomes modulated through the SWR1 complex has been suggested to underlie transcriptional regulation of thermal response in plants [23]. In fact, a direct measurement of transcriptional rates suggested that there exists a global transcriptional process modulating mRNA abundance by temperature [25]. However, the presence of H2A.Z in the gene body accounted for only part of this, suggesting that other factors contribute to the modulation of plant growth responses to ambient temperature variation.

In this thermo-sensory transcriptional network, the *PHYTOCHROME INTERACTING FACTOR 4 (PIF4)* has been suggested to be a central hub [18,20,26]. It has been shown that elevated ambient temperature leads to an increase in auxin levels, which in itself is under the control of *PIF4* [18,20,26]. Higher temperatures induce flowering and this process has been suggested to be mediated through *PIF4*, in addition to other known flowering time related genes [21,27,28]. Finally, altered regulation of the circadian clock has also been suggested to play a role in governing the plant growth in different temperatures (thermo-sensory growth response) [29]. The evening complex night-time repressor comprised of *EARLY FLOWERING 3* (*ELF3*), *ELF4* and *LUX ARRYTHMO1* (*LUX*) is inhibited by higher temperatures, which in turn can regulate the *PIF4* gene, modulating thermal response [29]. Thus an overarching theme that appears to emerge from these studies is that the thermal response in plants mostly occurs at a transcriptional level.

Furthermore, natural populations of *Arabidopsis thaliana (A*. *thaliana)* exhibit extensive variation in diverse traits including thermo-sensory growth and developmental responses [30]. The analysis of such natural variation has been very useful in identifying new mechanisms involved in the regulation of development by temperature, as illustrated with our current understanding of the vernalization process [17]. The first analyses of natural variation for growth processes in relation to high ambient temperature have already identified novel factors such as the *ISOPROPYL MALATE ISOMERASE LARGE SUB UNIT1* (*IIL1*) and the *ERECTA* genes [3,31]. In addition, natural variation in thermal response for flowering time has identified *FLOWERING LOCUS M* (*FLM*) as a regulatory factor, whose further analysis has suggested a role for *FLM* alternative splicing in the modulation of flowering by ambient temperature [21,32,33]. Thus our understanding of the molecular mechanisms and pathways that govern natural variation in thermo-sensory growth responses in plants is just beginning to emerge.

In this study, we have undertaken a natural variation approach and discovered that the uncharacterized and universally present gene, *ICARUS1* (*ICA1*), is required for plant growth specifically at high growth temperatures. Plants carrying loss-of-function alleles of *ICA1* are severely reduced in growth at high temperatures, but resume growth when reverted to lower thermal regimes. *ICA1* encodes a member of the tRNA^His^ guanylyl transferase (Thg1) superfamily [34]. The Thg1 superfamily has been of biochemical interest as its members share a striking structural similarity to nucleic acid polymerases and catalyze the addition of a guanosine residue to the 5’ end of the tRNA^His^, in an unusual 3’-5’ phosphodiester bond formation, which is required for tRNA^His^ maturation [34, 35]. However, their biological impact at the organismal level remains to be described. We have characterized the first plant member of this important class of proteins, demonstrating its biological role at cellular and organismal levels. In addition, we reveal substantial alternative splicing at the *ICA1* locus, which is associated with phenotypic suppression, indicating that alternative splicing can buffer the potential negative effects of some natural mutations. Together, our results show that allelic variation of a Thg1 superfamily gene contributes to the natural variation in the thermo-sensory growth response of plants.

## Results

### Sij-4 and Don-0 accessions display conditional and reversible growth phenotypes depending on ambient temperature

**Fig 1:**
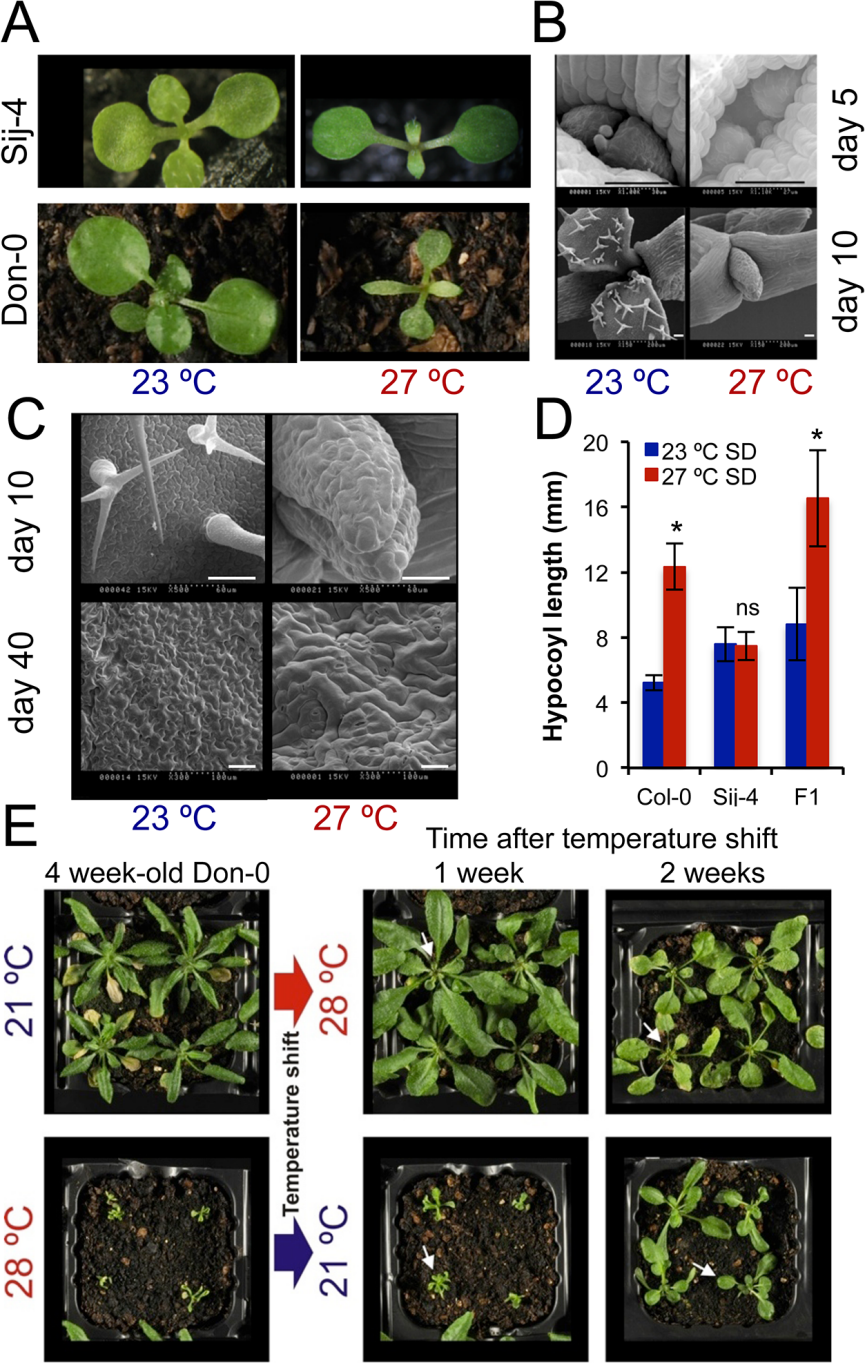
*ICA1 g*rowth phenotypes in natural accessions of Arabidopsis depend on temperature. (*A*) 2 week-old Sij-4 and Don-0 plants grown at 23°C and 27°C. (*B & C*) Scanning electron microscopy of Sij-4 grown at 23°C and 27°C. Differences in cell morphology are visible upon high magnification (*C*) from day 10. Scale bar = 50μM. (*D*) Hypocotyl elongation at 23°C and 27°C under short days (SD) in Col-0, Sij-4 and F_1_ plants derived from a cross between these two strains. 15–30 plants per genotype were analyzed for hypocotyl length measurement. *: *p*<0.0001; ns: not significant in Student t-tests comparing growth at the two temperatures. (*E*) Reversibility of the growth phenotype of Don-0 adult plants (4 week-old) in temperature shift experiments between 21 and 28°C. White arrows indicate new leaves developed after temperature shift.

To identify factors required for plant growth at high ambient temperatures, we grew a worldwide collection of *A*. *thaliana* accessions at standard (21–23°C) and high temperatures (27–28°C) and screened for accessions with severe growth defects at higher temperatures. We identified the Sij-4 and the Don-0 strains to be highly temperature-sensitive (Fig 1A). At high temperatures, they both had altered leaf morphology and reduced expansion of leaf blades (Fig 1A). The outgrowth of the first true leaf primordium was delayed and the cell morphology was also affected (Fig 1B and 1C). The high temperature-induced hypocotyl elongation was not observed in Sij-4 indicating a general impairment of growth at high temperatures (Fig 1D). Plants grown at intermediate temperatures displayed milder phenotypes indicating a quantitative nature of the growth defects (S1A Fig). In addition, these phenotypes were reversible on thermal shifts, although reversion was restricted mainly to the newly developed leaves, which initiated or stop growth after shifting to 21 or 28°C, respectively (Fig 1E). However, when Don-0 plants were transferred from 28 to 21°C, the oldest existing leaves did not grow, whereas younger existing leaves grew abnormally, leading to deformed leaves. Similar phenotypes were displayed by the two accessions throughout development with adult plants showing very reduced growth, smaller, pale and serrated leaves with reduced expansion of the leaf blade, altered phyllotaxy, plant architecture and severely impaired seed production (S1A–S1C Fig). Analyses of F_1_ and F_2_ populations derived from inter-crossing Sij-4 and Col-0 indicated that the growth defects were recessive and monogenic (280/1080 plants with growth defects, Chi-square p = 0.482; Figs 1D and S1E and S2). Further analysis of hypocotyl elongation in the F_2_ progeny revealed a highly significant genetic correlation between the leaf and hypocotyl phenotypes (*r*_*G*_^2^ = 0.88, *p*<0.0001) suggesting a shared genetic basis (S2B Fig).

### Positional cloning of *ICARUS1* identifies a universal protein required for plant growth at high temperatures

**Fig 2:**
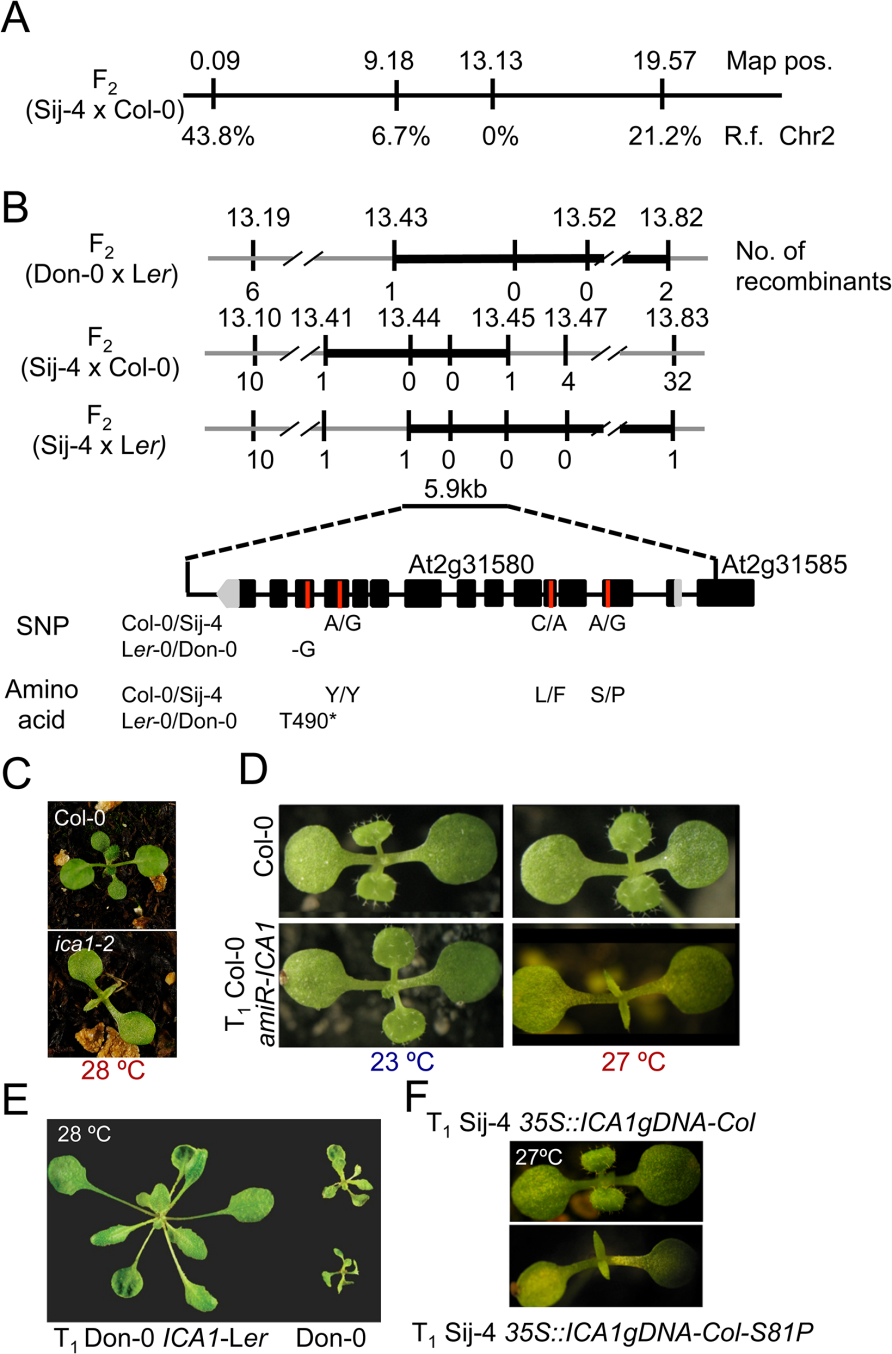
Positional cloning of *ICA1*. (*A*) Whole genome scanning of F_2_ (Sij-4 x Col-0) along with marker positions and recombination frequencies (R.f.) with *ICA1*. Map positions are given in Mb. (*B*) Fine mapping of *ICA1* in F_2_ (Don-0 x L*er*-0), F_2_ (Sij-4 x Col-0) and F_2_ (Sij-4 x L*er*-0) segregating populations. Map positions and the number of recombinants are indicated above and below the line respectively. An overlapping interval of 5.9 kb contains a single gene. The single nucleotide deletion of Don-0 and the missense mutations between Col-0 and Sij-4 along with the corresponding amino acid changes are given below. (*C*) Phenotype of the Col-0 and *ica1-2* (Col-0 background) grown at 27°C. (D) Phenotype of *35S*::*amiR-ICA1* in Col-0 background at 23°C and 27°C. (*E*) Phenotypic complementation of Don-0 with an *ICA1*-L*er* transgene at 28°C (4 week-old plants). (*F*) Transgenic suppression of *ICA1*-Sij-4 phenotype at 27°C by *35S*::*ICA1gDNA-Col* and lack of phenotypic suppression with the S81P mutation.

To identify the underlying genetic loci, we first analyzed F_1_ plants derived from a cross between the two sensitive accessions, Don-0 and Sij-4, which failed to complement each other indicating a common causal locus (S1D Fig). Consistent with this, genetic mapping using Don-0 x L*er* and Sij-4 x Col-0 F_2_ populations identified an overlapping region located in the middle of chromosome 2 associated with the phenotype (Fig 2A and 2B). To reflect temperature sensitivity, we named the presumed causal locus within this region *ICARUS1* (*ICA1*, after the Greek mythological character, who flew too close to the sun with wings attached with wax that melted at high temperature). Additional fine mapping using F_2_ populations derived from Sij-4 x Col-0 and Sij-4 x L*er* crosses, located *ICA1* in a 37 or 393 kb genomic region, respectively (Fig 2B). The two mapping intervals overlap in a 5.9 kb region that contains a single annotated gene, At2g31580. Two T-DNA insertion lines in At2g31580 (designated as *ica1-1* and *ica1-2*) in Col-0 background also displayed pleiotropic growth defects depending on temperature similar to Sij-4 and Don-0, although the exonic insertion line *ica1-2* showed stronger phenotypes (Figs 2C and S3A–S3G). Crosses between *ica1-1* and Sij-4 failed to complement the pleiotropic growth phenotypes, indicating that At2g31580 is *ICA1* (S3E Fig). This was further supported by transgenic lines carrying an artificial microRNA against At2g31580 in the Col-0 background, which displayed the *ica1* phenotype only at high temperatures (Figs 2D and S1F–S1H). Finally, the *ica1* phenotype was complemented in transgenic lines carrying the genomic sequence encompassing the *ICA1* locus from either L*er (ICA1gDNA*-L*er)* or Col-0 *(ICA1gDNA*-Col) driven by either the endogenous promoter (in Don-0 or *ica1-2* background) or the *35S CaMV* promoter (in Sij-4 background) confirming that At2g31580 is the *ICA1* gene (Figs 2E and 2F and S3H).

*ICA1* encodes a universal protein belonging to the tRNA^His^ guanylyl transferase (Thg1) super-family [34,36]. In contrast to human/yeast orthologs, *ICA1* encodes two tandemly repeated units of Thg1 within the protein and is known to be nuclear localized [37] (S4A Fig). To find out whether structural or regulatory polymorphisms result in Don-0 and Sij-4 loss-of-function alleles, we analyzed their *ICA1* sequence and expression levels. We did not find any obvious differences in *ICA1* expression levels across the accessions at lower temperatures, although there was a slight increase in *ICA1* expression of Sij-4 at higher temperatures (S5A and S5B Fig). Moreover, sequence analysis revealed a single 1 bp deletion in exon-12 of Don-0 *ICA1*, which is predicted to truncate the C-terminal half (position T490*, Fig 2B). Also, the Sij-4 strain harbors two nonsynonymous substitutions, of which the serine to proline (S81P) substitution corresponds to the nucleotide-binding site in the yeast homologue (equivalent to H34)[38] (Figs 2B and S4B). To test if this substitution affects *ICA1* function, we generated Sij-4 transgenic lines carrying a *35S*::*ICA1gDNA-Col* construct with an S81P substitution. Contrary to *35S*::*ICA1gDNA-Col* original construct, this transgene was unable to complement Sij-4 growth defects (Fig 2F), indicating that S81P is the causal polymorphism. The locations of both structural mutations in Don-0 and Sij-4 further suggest that the two ICA1 halves are required for its function and the structural perturbations might affect either the activity or the stability of ICA1 protein.

### Transcriptional and computational analyses suggest impairment of cell cycle in Sij-4

In order to understand the developmental mechanisms that lead to the growth defects observed in Sij-4, we analyzed genes that are differentially expressed between Col-0 and Sij-4 at different temperatures using RNA-seq analysis. In agreement with the temperature-dependence of the Sij-4 phenotype, more genes were differentially expressed between Col-0 and Sij-4 at 27°C than at 23°C (5449 versus 1661; S1 and S2 Tables). To assess whether the gene expression is specifically affected at higher temperatures or the differences in expression levels are more pronounced, we compared the fold changes in gene expression between Col-0 and Sij-4 at 23°C with the same genes at 27°C. There was a significant correlation between the fold changes, suggesting that the differences in expression levels were exacerbated at 27°C consistent with the quantitative nature of the observed phenotypes (S6 Fig). Of the 4236 genes differentially expressed only at 27°C (S3 Table), 2723 (65%) were down regulated in Sij-4 compared to Col-0. Gene ontology analysis [39,40] detected a highly significant enrichment of genes associated with cell proliferation, cytokinetic processes and DNA replication suggesting alterations of cell cycle regulation (S4 and S5 Tables). A comparison with a previously published analysis [41] revealed that the down regulation of gene expression in Sij-4 is more evident in genes expressed in the S and M phases of the cell cycle (S6 Table).

Since mutations in tRNA^His^ guanylyl transferase will possibly affect the availability of amino acyl histidine tRNA to be incorporated into proteins, we reasoned that proteins with higher number of histidine residues are more likely to be affected by mutations in *ICA1*. Accordingly, we undertook a computational analysis of the proteome of *A*. *thaliana* to identify processes enriched among proteins with relatively high histidine content, which revealed a significant enrichment for genes associated with cell cycle processes (S7–S9 Tables). Therefore, we hypothesized that a translational disruption of these proteins and the transcriptional down-regulation of cell cycle genes could confer the growth defects caused by the *ICA1* loss-of-function alleles.

### *ICA1*-Sij-4 loss-of-function alleles lead to a block in G2/M transition at high ambient temperatures

**Fig 3:**
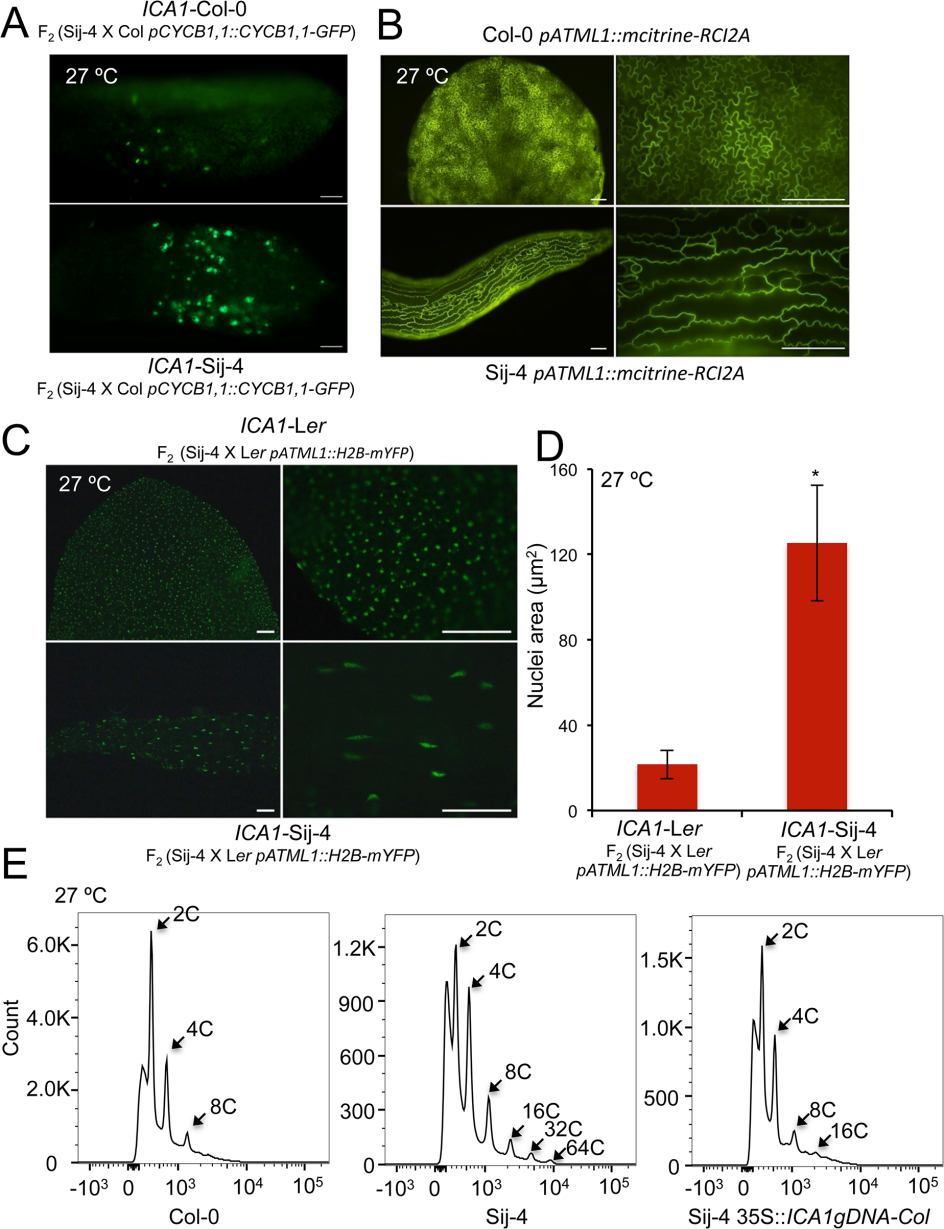
*ICA1* affects cell cycle and endoreduplication. (*A*) Expression pattern of Cyclin B1;1 in leaves of *ICA1*-Sij-4 and *ICA1*-Col plants segregating in an F_2_ (Sij-4 x Col-0) population grown at 27°C and analyzed using a pCycB1;1::CycB1;1-GFP marker. (*B*) Shape of abaxial epidermal cells of the first leaf from Col-0 and Sij-4 plants grown at 27°C and visualized using the plasma membrane marker *(pATML1*::*mCitrine-RCI2A)*. Magnification: left 10X, right 40X. (*C*) Epidermal cell nuclei of the first leaf of *ICA1*-Sij-4 and *ICA1*-L*er* plants selected in an F_2_ (Sij-4 x L*er*) family grown at 27°C and visualized with the histone H2B marker (pATML1::H2B-mYFP). Magnification: left 10X, right 40X. (*D*) Quantification of nuclei sizes measured as mean (± standard deviation) nuclei area in *ICA1*-Sij-4 and *ICA1*-L*er*-0 plants selected in an F_2_ (Sij-4 x L*er*-0) family grown at 27°C. *: *p*<0.0001 in Student t-test comparing nuclei sizes of both genotypic classes. (*E*) Flow cytometry analysis of Sij-4 plants compared with Col-0 and *35S*::*ICA1gDNA-Col* in Sij-4 background at 27°C. Scale bars in *A*, *B* and *C* are 100μM.

To further evaluate the potential impairment of the cell cycle, we tested if the Sij-4 growth phenotypes result from defects in cell proliferation using specific cell cycle markers. To this end, we used a marker line expressing the CyclinB1,1::GFP fusion protein driven by CycB1,1 promoter, which is known to be expressed only during the G2/M transition checkpoint and degraded at the end of the M phase [42–44]. Analysis of a segregating F_2_ population derived between *pCycB1;1*::*CycB1;1-GFP* line in Col-0 background and Sij-4, showed that CyclinB1,1-GFP expression was stronger in plants exhibiting the loss-of function mutant phenotype (henceforth referred to as *ICA1*-Sij-4) compared to normal appearing plants (henceforth referred to as *ICA1*-Col-0) (Fig 3A), which suggests that a fraction of *ICA1*-Sij-4 cells may be arrested in the G2/M transition phase. In agreement with this, RNA-seq analysis showed that 82% (67 out of 82) of the genes with expression that peaked at the G2/M boundary [45] were significantly down regulated in Sij-4 compared to Col-0 at high temperature (S10 Table). Cells arrested in the G2/M transition often undergo endocycling, and the timing of this arrest correlates with changes in cell size [46]. Consistent with this, we observed enlarged cells in Sij-4 plants using the pATML1::mCitrine-RCI2A plasma membrane marker [46] (Fig 3B). In addition, we used a histone H2B marker [46] (pATML1::H2B-mYFP) to visualize *ICA1*-Col-0 and *ICA1*-Sij-4 nuclei in abaxial epidermal cells, revealing larger elongated nuclei suggestive of increased DNA content (Fig 3C and 3D). This was verified by flow cytometric analyses, where Col-0 plants showed more than 90% of the cell nuclei with either 2C or 4C DNA content (Figs 3E and S7). By contrast, Sij-4 plants grown at high temperatures also contained nuclei with 32C and 64C at the expense of the 2C and 4C cells, which indicates that a significant proportion of the cells have gone through additional DNA replications (Figs 3E and S7). Introduction of the *ICA1*-Col-0 allele driven by the 35SCaMV promoter *(35S*::*ICA1gDNA-Col)* in Sij-4 suppressed this endoreduplication, hence indicating that it is caused by *ICA1* allelic variation (Figs 3E and S7). Thus, the growth defects observed in loss-of-function alleles of *ICA1* at high temperature are associated with disruptions in cell cycle regulation.

### *ICA1*-Sij-4 cells are hypersensitive to DNA damage

**Fig 4:**
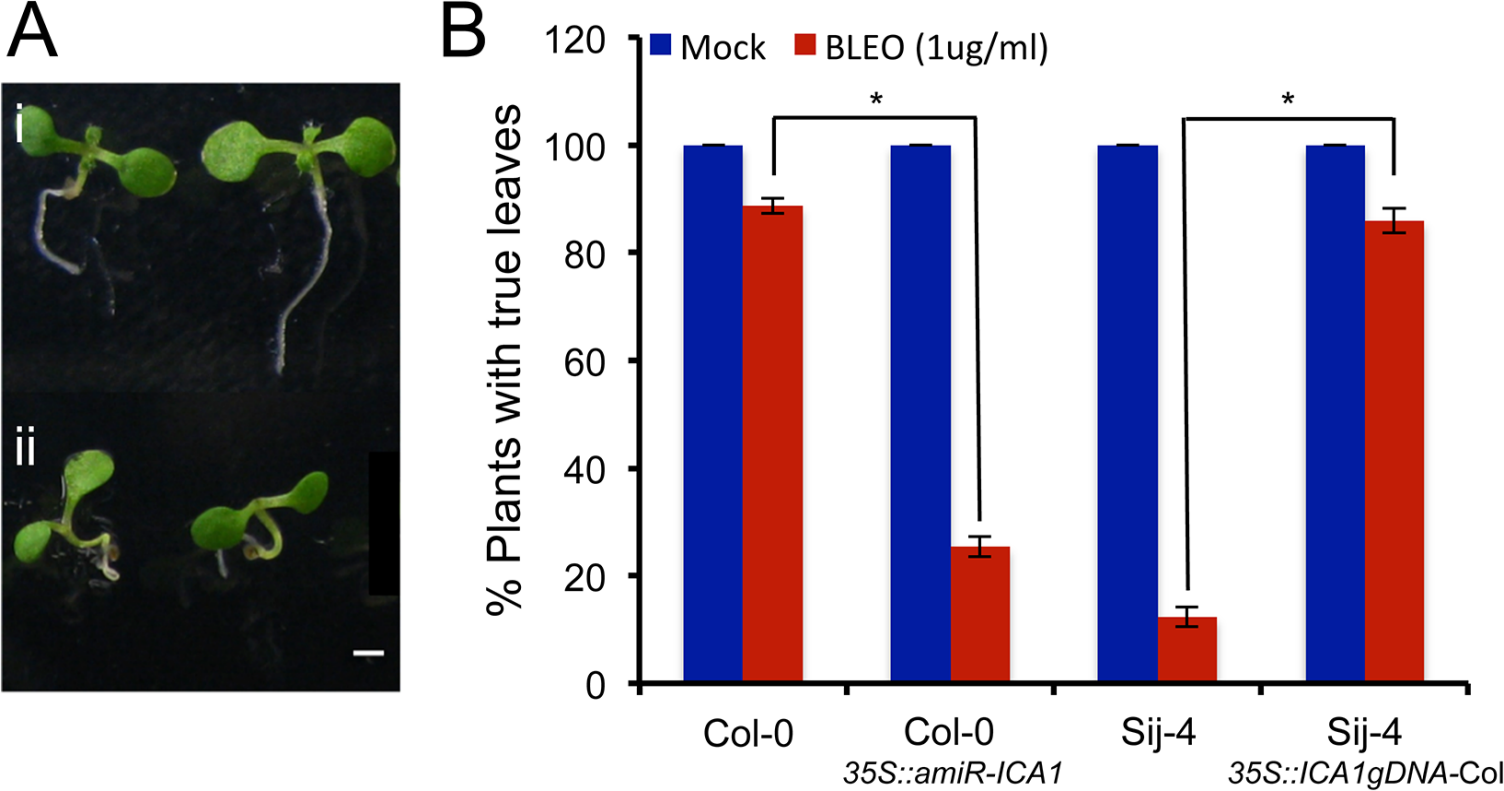
Sij-4 plants are hypersensitive to DNA damage. The sensitivity to DNA damage was assessed by the emergence of first leaves after Bleomycin treatment, which introduces double strand DNA breaks. (*A*) First leaf development in 10-day-old Col-0 seedlings grown at 23°C under long days. Examples for Sij-4 plants with (i) and without (ii) true first leaves are shown. (*B*) Percentage of plants developing true leaves when treated with Bleomycin (BLEO) or mock treated. Results from three independent experiments are shown for plants of Col-0, Sij-4, *35S*::*ICA1gDNA-Col* in Sij-4 background and *35S*::*amiR-ICA1* in Col-0 background. Error bars indicate standard deviation from three biological replicates with 50 to 100 seedlings each. *: *p*<0.0001 in Student t-tests comparing pairs of genotypes.

The G2/M cell cycle checkpoint allows the repair of DNA after DNA synthesis and it has been shown that DNA damage can induce endoreduplication in Arabidopsis [47,48]. Since at 27°C, a fraction of *ICA1*-Sij-4 cells were arrested at the G2/M transition coupled with endoreduplication, we tested if *ICA1* loss-of-function alleles affect the capacity of the plants to respond to DNA damage. To this end, we used the first leaf assay, which is based on the growth arrest of the first true leaves when subjected to DNA damage during early plant development [49,50]. Seedlings of Sij-4, Col-0, *35S*::*ICA1gDNA-Col* lines in Sij-4 background and *35S*::*amiR-ICA1* in Col-0 background were subjected to DNA damage by Bleomycin treatment and evaluated for their subsequent leaf development at 23°C (Fig 4A). Sij-4 seedlings were hypersensitive to Bleomycin with more than 80% of the plants lacking the first leaves compared to 15% in Col-0. The Sij-4 hypersensitivity was reduced in the *35S*::*ICA1gDNA-Col* lines in Sij-4 background, while *35S*::*amiR-ICA1* lines in Col-0 background displayed enhanced sensitivity to Bleomycin (Fig 4B). These results suggest that *ICA1* is also affecting the capacity to repair DNA damage, even under standard growth temperatures.

### Natural *ICA1* loss-of-function alleles are rare at global and regional scales but occur at high frequency in some local populations

**Fig 5:**
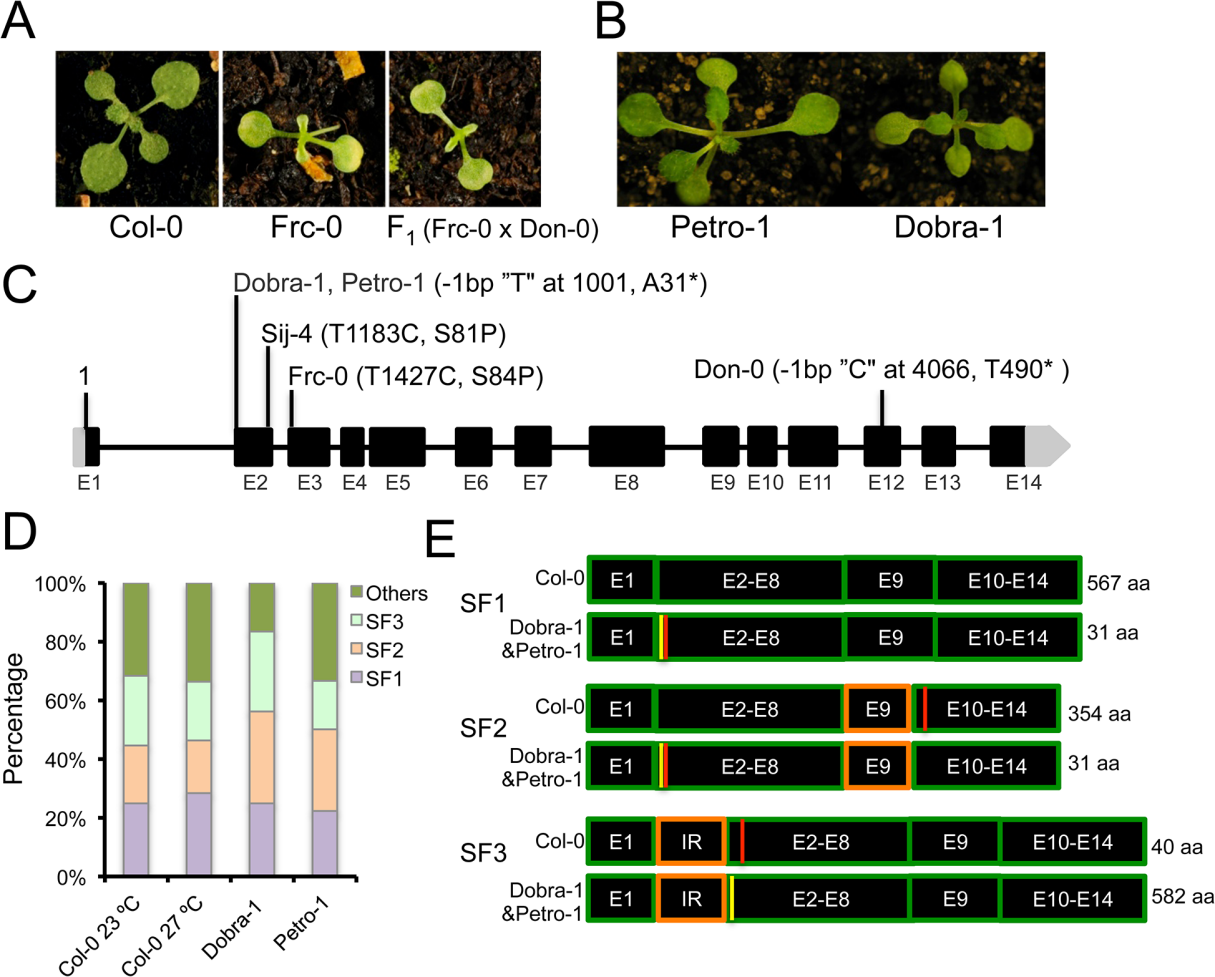
*ICA1* allelic variation and intragenic suppression by alternative splicing. (*A*) Frc-0 displays the growth defect at 27°C and the phenotype of F_1_ (Frc-0 x Don-0) plants demonstrates that *ICA1*-Frc-0 is another *ICA1* loss-of-function allele. (*B*) Absence of the growth defects in Petro-1 and Dobra-1 accessions grown at 27°C. (*C*) Natural polymorphisms of major effect observed in *ICA1* and their genomic positions. (*D*) Proportions of alternatively spliced transcripts of *ICA1* at 23°C and 27°C in Col-0 and at 27°C in Dobra-1 and Petro-1. (*E*) Schematic representation of the three major splice forms (SF1, SF2 and SF3), along with the predicted stop codons (shown as red lines) in Col-0 and Dobra-1/Petro-1. The single nucleotide deletion of Dobra-1/Petro-1 is shown as a yellow line, whereas the region affected by alternative splicing is marked in orange color. The predicted protein lengths are indicated in the right side of panel. SF2 is due to an alternative splice acceptor site for I8 in E9 resulting in a shorter E9 exon; SF3 is due to an alternative splice acceptor site in the first intron resulting in a partial intron retention (IR).

To assess the population frequency of *ICA1*-mediated growth alterations in response to temperature, we carried out additional phenotypic screening of a well-characterized regional collection of wild accessions from the Iberian Peninsula, where the accession Don-0 was originally isolated [51] (S8 Fig). Thus, we identified the Frc-0 strain displaying the temperature-dependent growth defects of Don-0 and Sij-4 (Fig 5A). F_1_ plants, derived from an Frc-0 x Don-0 cross, showed similar phenotypes to parental lines, indicating that Frc-0 carries an *ICA1* loss-of-function allele (Fig 5A). Sequence analysis identified a different SNP in the *ICA1*-Frc-0 allele, which causes a serine to proline substitution at position 84 (S84P). Since this serine is highly conserved in most plants and animals and is located close to the similar S81P polymorphism of Sij-4, it is likely that this structural mutation causes *ICA1* loss-of-function (Fig 5C). Therefore, we found a total of three different natural *ICA1* loss-of-function alleles from geographically distant locations (S8 Fig). To evaluate if these natural *ICA1* mutations might be tolerated at local population level, we also analyzed allele frequencies in Don-0 and Frc-0 populations from the Iberian Peninsula. Phenotypic and genotypic analyses of several individuals per population showed that Don and Frc locations differed in the amount genome-wide genetic diversity per population (gene diversity of 0 and 0.05 respectively) and were highly differentiated (average number of allelic differences of 0.33±0.02). However, the *ICA1*-Frc-0 allele is nearly fixed in Frc location (6 homozygous *ICA1*-Frc-0 out of 6 individuals) and the *ICA1*-Don-0 allele was maintained at high frequency over time, since 7 out of 9 individuals were homozygous for *ICA1*-Don-0 allele six years after the initial Don collection [52].

### Natural intragenic suppression of mutations through alternative splicing

As a complementary approach to find additional natural *ICA1* loss-of-function alleles, we analyzed *ICA1* sequences from the Arabidopsis 1001genome project, which recovered the S81P and T490* mutations in Sij-4 and Don-0 respectively. In addition, an identical single bp deletion was found in the Petro-1 and Dobra-1 accessions. This mutation is predicted to shift the reading frame and generate a short truncated protein of 31 amino acids (Fig 5C). While we confirmed this deletion through Sanger sequencing, Petro-1 and Dobra-1 failed to display the described phenotypes associated with *ICA1* loss-of-function, suggesting the presence of modifiers (Fig 5B). To assess whether intragenic second-site suppression could account for the lack of disrupted phenotypes in Petro-1 and Dobra-1, we compared *ICA1* cDNAs from these strains with those of Col-0. We first cloned and sequenced Col-0 cDNAs isolated from 23°C (76 clones) or 27°C (95 clones), which identified a total of 22 different transcripts that uncovered extensive alternative splicing at *ICA1* (S11 Table). Three splice forms, referred to as SF1, SF2 and SF3, accounted for 70% of transcripts (Fig 5D and S11 Table) and no significant difference was found in the frequency of *ICA1* transcripts between the two temperatures (S11 Table and Fig 5D). Only the SF1 transcript encodes the full length ICA1 protein of 567 amino acids, whereas transcripts SF2 and SF3 are predicted to generate truncated proteins of 354 or 40 amino acids respectively (Fig 5E). Since the truncated proteins are smaller than that encoded by Don-0 strain, these are likely to be nonfunctional. SF3 results from the use of an alternative splice acceptor site in intron 1 leading to the retention of 46 bp, which is predicted to shift the reading frame and to generate a premature stop codon in exon 2 (Fig 5E). Sequencing of cDNAs from Dobra-1 (48 clones) and Petro-1 (18 clones) revealed a splicing pattern similar to Col-0 (S11 Table). The single nucleotide deletion of Dobra-1/Petro-1 leads to a premature stop codon that would result in a truncated protein of 31 amino acids in transcripts encoded by SF1 and SF2. However, this deletion restores the SF3 open reading frame, resulting in a protein similar to that encoded by Col-0 SF1 with the addition of 15 amino acids in the N-terminal extension of ICA1 (S9 Fig and S11 Table). Thus, alternative splicing enables the natural intragenic suppression of an otherwise *ICA1* loss-of-function allele.

## Discussion

### Modulation of fundamental processes related to protein biosynthesis might regulate thermo-sensory growth responses in plants

Temperature regulation of plant growth and development has been described until now mostly in the context of transcriptional regulation [14,23]. However, in this study we have demonstrated that *ICA1*, which encodes a member of the universally present Thg1 superfamily that is known to be involved in the tRNA^His^ maturation [34–36], is required for normal plant growth specifically at high ambient temperatures. Consequently, *ICA1* appears to be an essential factor that is necessary for the regulation of thermo-sensory growth responses in *A*. *thaliana*. Both, natural and induced, *ICA1* loss-of-function alleles show strong temperature-sensitive pleiotropic effects on plant growth throughout vegetative and reproductive development. These pleiotropic phenotypes are consistent with a function for *ICA1* in a basic molecular process like the tRNA^His^ maturation mediated by Thg1 proteins. In agreement with this *ICA1* function, our results suggest that most growth defects caused by *ICA1* loss-of-function alleles could be due to indirect *ICA1* effects on proteins involved in cell cycle processes and containing high histidine. In addition, the quantitative and reversible nature of the growth defects caused by *ICA1* loss-of-function alleles further suggest the precise modulation of this fundamental biological process depending on temperature. Interestingly, a similarly strong temperature-sensitive growth phenotype has been previously shown in the Bur-0 strain to be caused by a mutation in the *IIL1* locus that encodes an enzyme involved in leucine biosynthesis [3]. Therefore, thermal responses in Arabidopsis growth are likely regulated not only by direct transcriptional regulation, but also by modulation of other fundamental biological processes related to the general regulation of protein biosynthesis.

### Thg1 family members share a conserved role in cell cycle regulation

We have characterized at the cellular and organismal level the first plant member of the highly conserved Thg1 superfamily, *ICA1*, showing its crucial developmental effects in *A*. *thaliana*. The strong *ICA1* pleiotropic effects found at the organism level appear determined by its effect at the cellular level, since *ICA1* is required for plant cell cycle progression and cell division. Consistent with a role in growth rather than cellular differentiation, the cell division disruption caused by *ICA1* did not affect the general pattern of vegetative and reproductive organs, but mostly alters their sizes. The yeast homologue of ICA1 (Thg1p) was identified as a protein interacting with the replication origin recognition complex, and defective alleles led to defects in cell division similar to those that we have found in Arabidopsis [53]. In addition, the human homologue, referred to as ICF45, was isolated in a cDNA library screen as a factor expressed in a cell-division dependent manner [54]. Together these findings indicate that Thg1 proteins display a conserved function in cell division in most eukaryotes, from uni- to multicellular organisms.

Despite the primary molecular function described for Thg1 proteins are to provide mature tRNA^His^ [36], several observations have suggested additional potential functions. First, the Thg1 proteins are also present in organisms in which the 5’ G is already encoded in their genome, suggesting that this enzyme may have another ancestral function [34]. Second, the Thg1 proteins share a striking structural similarity with nucleic acid polymerases [38,55,56]. Third, they are unique in their ability to use both NTPs and dNTPs as substrates in the 3’-5’ polymerization reaction that they catalyze [57]. Fourth, Thg1 proteins have been shown to interact with the origin recognition complex, which has a primary role in DNA replication [53]. Based on these observations, it has been suggested that Thg1 proteins may also have a function in DNA/RNA repair [34,58]. In agreement with this hypothesis, *ICA1* loss-of-function alleles display hypersensitivity to DNA damage. Hence, further investigations on the potential role of Thg1 superfamily in DNA/RNA repair and its link to cell cycle regulation are warranted.

### Allelic variation at *ICA1* accounts for natural variation in thermo-sensory growth responses of *Arabidopsis thaliana*

The characterization of Arabidopsis wild accessions revealed several instances of temperature-sensitive growth arrest, which is largely determined by *ICA1* allelic variation. Natural loss-of-function alleles of *ICA1* have arisen multiple times independently, since we found three different *ICA1* natural loss-of-function mutations in global and regional collections from distinct geographic locations. Overall, the low frequency of such alleles (3 out of more than 300 analyzed strains) and their restriction to individual populations, suggest that *ICA1* loss-of-function is deleterious in most natural environments and locations. This is further supported by the buffering of a potentially deleterious mutation through alternative splicing found in Petro-1 and Dobra-1 accessions. However, *ICA1* loss-of-function alleles show high frequency at the local population level, thus suggesting that they are neutral in these populations. Therefore, this cryptic genetic variation at *ICA1* is, most likely, conditionally neutral under natural conditions. Nevertheless, the quantitative and reversible nature of the temperature dependency, both at the organism phenotypic level and at the level of gene expression, as well as the presence of splice variants that encode potentially non-functional proteins in significant proportions (~70%, S11 Table), also suggest that *ICA1* effects might be regulated in a quantitative manner to modulate plant growth in relation to temperature. Accordingly, we speculate that the fine tuning of plant growth by the reversible growth arrest caused by natural *ICA1* loss-of-function alleles might reflect an alternative mechanism to respond to abiotic stress, which could be locally advantageous under certain unfavorable high temperatures.

### Gene duplication may account for the temperature conditionality of *ICA1* phenotypes

Even though *ICA1* is required for growth at high ambient temperature but not at standard temperature, the precise mechanism accounting for the temperature sensitivity of the phenotypes caused by *ICA1* loss-of-function alleles remains unknown. It is possible that this conditionality is an indirect effect derived from the temperature regulation of any downstream molecular component that is necessary for *ICA*1 effects on cell division. Alternatively, the temperature sensitivity of *ICA1* effects might be the result of functional divergence of Thg1 proteins, because *A*. *thaliana* carries two genes encoding members of the Thg1 superfamily that may act redundantly [37]. Most likely, this duplication accounts for the viability of *ICA1* loss-of-function genotypes, in comparison with the lethality of null alleles in the single copy *Thg1* gene of yeast [36]. However, the strong phenotypes of *ICA1* loss-of-function alleles at high temperature indicate that the two close paralogs are not fully redundant but show certain functional diversification. Future studies will further elucidate the relative contribution of functional divergence of plant Thg1 encoding genes to the temperature sensitivity of *ICA1* growth phenotypes.

## Materials and Methods

### Plant material and growth conditions

*A*. *thaliana* accessions Col-0, Sij-4, Petro-1 and Dobra-1 and the T-DNA insertions lines for At2g31580 (SALK035242 and Wisc DsLox Hs 036_12H, in intron 1 and exon 8, and referred to as *ica1-1* and *ica1-2*, respectively) were obtained from Arabidopsis Biological Resource Center. The Iberian collection used for the phenotypic screen has been previously described [51]. Frc-0 and Don-0 local populations were sampled in this study by collecting six and nine individuals respectively. Genetic diversity of the two populations was analyzed by genotyping a genome-wide set of 249 SNPs as previously described. [59].

Plants for phenotyping, crossing and propagation were grown on soil, at 23°C, in growth rooms under long days (LD, 16-h-light/8-h-dark cycles). Temperature dependent phenotypic analyses were done under short days (SD, 8-hr-light/16-hr-dark cycles) at 23°C and 27°C (for most of the experiments with Sij-4) or in long days at 21°C or 28°C (for most of the experiments with Don-0). For DNA damage assays and transcriptome analyses, sterilized seeds were plated on 0.5x MS media supplemented with 1% sucrose, and plates were placed in growth chambers (Percival Scientific, Canada) in SD at the required temperatures. For transformation, to accelerate flowering, Sij-4 was vernalized by imbibing seeds in water and placing them at 4°C in the dark for at least 4 weeks before planting.

For measurements of hypocotyl elongation in response to high temperature, F_2_ (Sij-4 x Col-0) seeds were grown at 23°C and 27°C in SD, after 2 days of stratification at 4°C in darkness. Two week-old F_2_ seedlings were then collected and photographed. Early leaf development phenotype was scored based on *ICA1* loss-of-function leaf phenotype and the hypocotyl length was measured with ImageJ64 for Mac.

### Positional cloning

F_1_ plants derived from reciprocal crosses between Sij-4 and Col-0 were tested at 27°C SD to evaluate *ICA1* phenotype. The initial mapping was performed using 96 plants with *ICA1*-Sij-4 phenotype collected from a F_2_ (Sij-4 x Col-0) population and 300 *ICA1*-Don-0 phenotype plants derived from a F_2_ (Don-0 x L*er*) family. For fine mapping we used 2500 and 1500 mutant plants respectively from F_2_ (Sij-4 x Col-0) and F_2_ (Sij-4 x L*er*) populations. Genetic markers used for fine mapping are given in S11 Table. Analyses of available sequences from Sij-4, Bur-0, C24, Col-0 and L*er*-0 (1001 genome project; http://1001genomes.org/) were performed to identify nucleotide polymorphisms in mapping intervals. The final mapping interval (5.9 Kb) was then fully sequenced in Sij-4 and a 5.3 Kb region containing the *ICA1* gene was sequenced in Don-0 to identify sequence variants.

### Generation of constructs and transgenic lines

For *ICA1* complementation in Sij-4, a construct containing 4.7 Kb genomic DNA encompassing the entire coding region of *ICA1* from Col-0 (*35S*::*ICA1gDNA-Col*) driven by 35S CaMV promoter was cloned into the Gateway entry vector pDONR207 and moved into pFK210 through LR reaction. Constructs in pFK210 were then electroporated into *Agrobacterium tumefaciens* GV3001 and transformed into Sij-4 plants by floral dipping [60]. Similarly, complementation of Don-0 and the *ica1-2* T-DNA mutant was done using a 5.3 Kb L*er* genomic fragment containing the complete *ICA1* coding region and the intergenic adjacent sequences cloned in the binary vector pCAMBIA3300.

To generate a construct containing the S81P mutation (*35S*::*ICA1gDNA*-Col-S81P), the *35S*:: *ICA1gDNA*-Col construct was used as template to introduce a T to C conversion (corresponding to TAIR10 position 13444655, Chr 2) by site-directed mutagenesis, according to QuikChange II Site-Directed Mutagenesis Kit (Agilent Technologies). The *35S*::*amiR-ICA1* construct was designed using primers listed in S12 Table according to Schwab et al [61] and cloned into pFK210 for transformation.

### Microscopy

To investigate the cell cycle of plants carrying *ICA1* loss-of-function alleles, a pCyclinB1;1::CyclinB1;1-GFP stable transgenic line in Col-0 background (kindly donated by Peter Doerner, University of Edinburgh, UK) was crossed with Sij4. Two week-old F_2_ young seedlings derived from that cross were dissected to obtain shoot apices and early leaves for fluorescence microscopy analysis. The pCyclinB1;1::CyclinB1;1-GFP signal was then observed by fluorescence microscopy and compared between plants showing *ICA1*-Sij-4 and *ICA1*-Col phenotypes. Nucleus and cell sizes of abaxial epidermal cells from the first pair of true leaves were measured using the nucleus marker pAR98 (*pATML1*::*H2B-mYFP*) and the plasma membrane marker pAR169 (*pATML1*::*mCitrine-RCI2A*)[46] respectively. Plasma membrane marker pAR169 was introduced by plant transformation in Sij-4 and Col-0 plants to compare between *ICA1*-Sij-4 and *ICA1*-Col cells. For nuclear marker pAR98, a stable transgenic line in L*er* background was crossed with Sij-4, and the F_2_ plants were used to compare between *ICA1*-Sij-4 and *ICA1*-L*er* cells. The nuclear area was measured manually using more than 15 nuclei per genotype using ImageJ. The surface of 5,10 or 40 day-old first true leaves was visualized using low-temperature SEM according to Feiler et al [62].

### Flow cytometry

For measurements of DNA content, 3 week-old plants of Col, Sij-4 and *35S*::*ICA1gDNA*-Col in Sij-4 background grown at 27°C SD, were chopped with a sharp razor, and the nuclei stained with propidium iodide according to the manufacturer’s protocol (CyStain UV Precise P; PARTEC). The distribution of DNA content in cell populations was then measured with a flow cytometry analyzer LRS IIb (BD Biosciences). The DNA contents of Sij-4 and *35S*::*ICA1gDNA*-Col in Sij-4 plants were compared against Col-0 and the significance was tested using a Chi-square test.

### DNA damage assays

The first leaf assay was performed to evaluate DNA damage response with Col-0, Sij-4, *35S*::*ICA1gDNA*-Col in Sij-4 background and *35S*::*amiR-ICA1* in Col-0 background grown under 23°C SD as described previously [50]. Briefly, four-day-old seedlings were treated with a DNA damage reagent, the radiomimetic drug Bleomycin (BLEO, EMD Millipore) and the development of the first two leaves was analyzed at early stage. Seedlings were transferred to liquid 0.5 MS medium at day 4, either with or without 1μg/ml or 2μg/ml of BLEO. At day 9, BLEO-treated seedlings were washed with plain liquid 0.5 MS media and transferred back to 0.5 MS plates. The phenotype of first true leaves was then scored 24 hours later at day 10.

### Analysis of *ICA1* expression and alternative splicing

Leaf tissue from 4 week-old plants grown at 23°C and 27°C under SD was collected and RNA was isolated with Trizol (Invitrogen). RNA was reverse transcribed using the Roche first strand cDNA synthesis kit (Roche). For *ICA1* expression, RNA was isolated from three week-old plants grown at 23°C and 27°C under long days analyzed by quantitative PCR using primers described in S12 Table. To analyze alternative splicing, cDNAs were amplified from Col-0, Petro-1 and Dobra-1 grown at 23°C or 27°C using full length *ICA1* coding region primers described in S12 Table, and cloned in to pGEM-T vector (Promega). Clones were sequenced with pUC/M13F and pUC/M13R primers (Macrogen, South Korea). After quality control, sequences were obtained for 48–96 clones as shown in S11 Table (76 and 96 colonies from Col-0 23°C and 27°C respectively; 48 colonies from Dobra-1 and 18 colonies from Petro-1 in 27°C short day). Sequences were aligned with Seqman (DNAStar Lasergene) to identify alternative splicing at 23°C and 27°C.

### RNA-seq transcript analysis

For transcriptome analysis, about one hundred 6-day-old seedlings of Col-0 or Sij-4 grown at 23°C or 27°C in growth chambers (GR-36, Percival Scientific, Canada) in SD were harvested around 1.00 PM (5 hours after the beginning of the light regime). The RNA was extracted using Isolate II RNA plant kit (Bioline Pty Ltd, Australia) and RNASeq was done with Illumina HiSeq2000 platform by BGI (BGI, China). Three and two biological replicates were used for Col-0 and the Sij-4 samples respectively. Analysis of differential expression between samples was conducted using the edgeR Bioconductor package [63]. Paired-end Illumina RNA-Seq reads of 90bp length were aligned to the TAIR10 reference Arabidopsis genome, using the Subread pipeline's subread-align program with its default parameters [64]. Raw abundance counts of each gene were subsequently produced by running the Subread pipeline’s featureCounts program on the SAM file produced in the previous step, using the TAIR10 Arabidopsis genome annotation file (downloaded from TAIR) and the-p and-R parameters to convert mapped reads to mapped RNA fragments (a pair of forward and reverse reads) and to output read counting results for each fragment, respectively. The resulting list of abundance counts for each gene was used as input data to the edgeR differential expression software. Differential expression was analyzed by the edgeR BioConductor package using the GLM (generalized linear model) approach, with replicate number added as a factor to the generalized linear model to mitigate for a batch effect. In case of Col-0, where three replicates were present, 'replicate 1' and 'replicate 2 or 3' were used as factors, since replicates 2 and 3 were produced at the same time and were not subject to a batch effect between the two. As per edgeR defaults, p-values for differential expression were adjusted for multiple hypotheses testing by Benjamini-Hochberg p-value correction. To analyze the correlation between changes in expression, log fold changes from the edgeR output of the differential expression between Col-0 and Sij-4 at 23°C and 27°C was plotted against each other and the Pearson correlation was calculated. There was no difference in Pearson correlation between genes that were down regulated or up regulated in Sij-4 suggesting that the observed differences are not associated with the polymorphisms between Sij-4 and Col-0 affecting the alignment of reads.

### GO enrichment analysis

The gene lists generated through the analysis of differential expression were used in the online program GOrilla to identify enriched GO terms [39] and the GOrilla output was summarized and visualized through REViGO [40]. In order to identify the Histidine rich proteins, we used three different approaches. First, we used a single list of the Arabidopsis proteins ranked by amino acid content (numbers) and analyzed through GOrilla and REViGO as described above. Second, we took the top 5% of the proteins with high amino acid content and compared this list using the whole proteome as the background through GOrilla and REViGO. Third, we removed the tail ends of the distribution taking only proteins that fell in between the 10^th^ and 90^th^ percentile in terms of the protein length and analyzed the top 5% of these through a similar analysis. The analysis was done with all amino acids, with all proteins ranked by length or with the top 5% of the largest proteins with the entire proteome as controls.

### Databases

The sequences of the *ICA1* from various strains and the sequences of different splice forms are available through Genbank accession numbers KP759903-KP759939. The transcriptome data has been submitted to the NCBI Sequence Read Archive and is available under the accession number SRP053394.

## Supporting Information

S1 Fig *ICA1* phenotypes of adult plants. (*A*) 5-weeks-old Don-0 plants grown under long-day at 23°C or 28°C and 9-week-old plants grown at intermediate 26°C. (*B*) Don-0 plants at reproductive phase grown at 21 and 26°C. (*C*) 8-weeks-old Sij-4 plants grown under short-day at 23°C or 27°C. (*D*) *ICA1*-Sij-4 phenotype of F_1_ plants derived from crosses between Sij-4 and Don-0 grown at 27°C. (*E*) 4-weeks-old F_1_ (Sij-4 x Col-0) plants displaying normal development at 27°C. (*F*). 4-weeks-old *35S*::*amiR-ICA1* plants in Col-0 background compared with Col-0 at 27°C. (*G*) Malformed siliques produced by *35S*::*amiR-ICA1* plants grown under long-day (LD) conditions. (*H*) *ICA1* expression level in 5 independent T1 *35S*::*amiR-ICA1* transgenic lines in Col-0 background, normalized to tubulin. The expression levels are shown relative to that in Col-0. Error bars indicate ± standard errors based on technical replicates derived from three independent cDNAs. ***: p<0.0001. Scale bars: *A*, *B* & *C* = 12mm; *D* & *F* = 5mm; *E* = 6mm; *G* = 1mm.
(TIF)

S2 Fig Genetic correlation between leaf and hypocotyl phenotypes in plants segregating for *ICA1*. (*A*) Distribution of hypocotyl length in F_2_(Sij-4 x Col-0) plants grown at 23°C and 27°C. (*B*) Distribution of hypocotyl length in F_2_(Sij-4 x Col-0) grown at 27°C and classified according to *ICA1* phenotype in leaves. The plants are color coded to differentiate the *ICA1-Sij-4* plants (blue) and *ICA1*-Col-0 plants (red). Short hypocotyls co-segregate with leaf growth defect.
(TIF)

S3 Fig T-DNA insertion lines in *ICA1*. (*A*) Schematic representation of the T-DNA insertions at the *ICA1* locus. (*B*) Confirmation of T-DNA insertion in *ica1-1*. Amplified products in Col-0 and *ica1-1* plants with left border T-DNA primer along with primers flanking the insertion site. M:I kb+ ladder (*C*) Expression of *ICA1* in 3 independent *ica1-1* plants compared with Col-0. (*D*) Phenotypes of *ica1-1* T-DNA line grown at different temperature and light conditions. The arrows indicate serrations in first leaves, which are not normally seen. Variable *ica1* phenotype is identified in *ica1-1* T-DNA lines indicating that this intronic insertion line is a partial loss-of-function allele. (*E*) The derived F_1_ plants between Sij-4 and *ica1-1* insertion line grown at 27°C SD. (*F*) and (*G*) Vegetative and reproductive phase phenotypes of *ica1-2* T-DNA line compared with Col-0 at 23°C or 27°C, respectively. (*H*) *ica1-2* insertion line and its transgenic complementation by *ICA1*-L*er* allele at 28°C.
(TIF)

S4 Fig ICA1 protein structure in different species. (*A*) Protein structure and homology between ICA1 from Arabidopsis and Thg1 from yeast and human. (*B*) Sequence alignment of the region containing S81P polymorphism in Sij-4 compared with Col-0 reference strain of *Arabidopsis thaliana* and the following species: *Oryza sativa*, *Apis mellifera*, *Drosophila melanogaster*, *Saccharomyces pombe*, *Saccharomyces cerevisiae* and *Homo sapiens*. Identical amino acids are shaded in black, while S81P, equivalent to yeast H34 and to the nucleotide binding site described in the human homologue [38] is marked in red color.
(TIF)

S5 Fig *ICA1* expression analysis in natural accessions. (*A*) Relative *ICA1* expression levels in L*er* and Don-0 grown at different temperatures under LD. (*B*) Relative *ICA1* expression levels in Col-0 and Sij-4 accessions and in *ICA1*-Sij-4 and *ICA1*-Col plants selected from a F_2_ (Sij-4 x Col-0) grown under SD at 27°C, based on leaf phenotypes. Mean expression levels ± standard deviations are shown. *ACTIN2* and *TUB2/3* were used as internal controls for expression analyses in A and B, respectively. L*er* and Don-0 did not differ statistically (ns: *p*>0.05), whereas differences between *ICA1*-Col and *ICA1*-Sij-4 were significant (*: *p*<0.05).
(TIF)

S6 Fig Correlation plots of fold changes in gene expression at 23°C and 27°C. Log fold changes in gene expression between Col-0 and Sij-4 at 23°C and 27°C are plotted against each other and the R^2^ for the correlations are shown for all genes that were detected to be expressed (All genes) or for genes that are detected to be differentially expressed between Col-0 and Sij-4 at 23°C (DE at 23°C) or 27°C (DE at 27°C). Negative and positive log fold values indicate lower and higher Sij-4 expression in relation to Col. All correlations are significant (p<0.0001) suggesting that the directionality of changes in gene expression remain the same across temperatures and the differences are more pronounced at one or the other temperature.
(TIF)

S7 Fig Flow cytometry analysis of DNA content. Proportion of nuclei with different DNA content in Col-0, Sij-4 and *35S*::*ICA1gDNA-Col* in Sij-4 background. Mean values ± standard deviations are shown. The *p*-values obtained through Chi-square analysis are shown above. ns: *p*>0.05; *:*p*<0.0001.
(TIF)

S8 Fig Geographic distribution of natural *ICA1* mutations. (*A*) Distribution of different *ICA1* alleles across the globe. (*B*) Location of populations with high frequency of *ICA1* loss-of-function alleles from the Iberian Peninsula.
(TIF)

S9 Fig Restoration of the *ICA1* reading frame in Dobra-1/Petro-1 allele. (*A*) Partial sequences of splice form 1 (SF1) and SF2 in Col-0 and Dobra-1/Petro-1 spanning the first intron and their predicted impacts on protein sequence. The protein sequence resulting from Dobra-1/Petro-1 allele, carrying a single bp (T) deletion that causes a frame shift, is shown in red. (*B*) Sequence of splice form 3 (SF3) in Col-0 showing the frame shift due to the partial intron retention and the corresponding protein sequence. The single bp (T) deletion of Dobra-1/Petro-1 restores the reading frame in SF3, which results in a protein similar to that encoded by SF1 with 15 additional amino acids. Intron sequences are shown in small blue letters and the additional amino acids are shown in red.
(TIF)

S1 Table List of genes detected to be differentially expressed between Col-0 and Sij-4 at 27°C at a false discovery rate of less than 0.05. logFC: log fold change; logCPM: log counts per million; LR: likelihood ratio; *p*-value: *p*-value for the differential expression; FDR: False Discovery Rate. A negative log fold change indicates that the gene is down regulated in Col-0 compared to Sij-4 and vice versa.
(XLS)

S2 Table List of genes detected to be differentially expressed between Col-0 and Sij-4 at 23°C at a false discovery rate of less than 0.05. logFC: log fold change; logCPM: log counts per million; LR: likelihood ratio; *p*-value: *p*-value for the differential expression; FDR: False Discovery Rate. A negative log fold change indicates that the gene is down regulated in Col-0 compared to Sij-4 and vice versa.
(XLS)

S3 Table List of genes detected to be differentially expressed between Col-0 and Sij-4 only at 27°C and not at 23°C at a false discovery rate of less than 0.05. logFC: log fold change; logCPM: log counts per million; LR: likelihood ratio; *p*-value: *p*-value for the differential expression; FDR: False Discovery Rate. Computational description of genes is also included. A negative log fold change indicates that the gene is down regulated in Col-0 compared to Sij-4 and vice versa.
(XLS)

S4 Table GO terms enriched in genes down regulated in Sij-4 at 27°C. *p*-values are for the enrichment not corrected for multiple testing. Only the GO-terms that gave *p*-values beyond the default threshold of <10^–3^ are given. FDR q-value is the correction of the above *p*-values using Benjamini and Hochberg correction. N: Total number of genes; B: Total number of genes associated with a specific GO term; n: Number of genes in the target list; b: number of genes in the intersection. Cell cycle associated GOs are highlighted.
(XLS)

S5 Table REViGO treemap output for GO terms. Only the GO-terms that gave *p*-values beyond the default threshold of <10^–3^ are used in this analyses. Table shows the output for genes that are down regulated in Sij-4 compared to Col-0 specifically at 27°C.
(XLS)

S6 Table Chi-square analysis of the expression changes among genes associated with different phases of cell cycle. Overlapping set of genes that showed differential expression between Sij-4 and Col-0 at 27°C with the published data from [41], was compared through Chi-square analysis. The chi-square value was calculated for each phase of the cell cycle by analyzing whether the up regulation/down regulation in Sij-4 deviates from the expectation based on the proportion of up regulated or down regulated genes in the total list of differentially expressed genes.
(XLS)

S7 Table GO terms enriched among proteins with high Histidine content based on a single ranked list of proteins sorted by their His content. FDR q-value is the correction of the above *p*-value using Benjamini and Hochberg correction. N: Total number of genes; B: Total number of genes associated with a specific GO term; n: Number of genes in the target list; b: number of genes in the intersection. Cell cycle associated GOs are highlighted.
(XLS)

S8 Table REViGO tree map output of the GO terms enriched in proteins with high Histidine content based on a single ranked list of proteins sorted by their His content. (XLS)

S9 Table Analysis of biological processes enriched among proteins with high content for each amino acid. Table shows the top three terms identified through REViGO analysis for GO terms enriched among proteins with high content of each amino acids. First column represents the enrichment of processes among the top 5% of proteins with high content for each amino acid compared with all proteins. The 5% largest proteins are used as a control. The second column represents the same analysis, but with a ranked list based on the content for each amino acid. A ranked list of all proteins based on the total length was used here as a control. The last column represents a similar analysis, but restricted for proteins within the 10^th^ to 90^th^ percentile based on their length. The cell cycle process is highlighted.
(XLS)

S10 Table Changes in gene expression among genes known to be peaking around the G2/M transition based on the data from [45]. logFC: log fold change; logCPM: log counts per million; LR: likelihood ratio, p-value: p-value for the differential expression; FDR: False Discovery Rate.
(XLS)

S11 Table Alternatively spliced forms of *ICA1*. Table shows *ICA1* splicing forms across different genotypes and temperatures. The actual number of clones and the corresponding percentages are given. The predicted lengths of the proteins in Col-0 (SF1 to SF22), Dobra-1 (SF24) as well as Petro-1 (SF1 to SF4) are included. The effect of temperature and accession was tested through nominal logistic regression with the splice variants (SF1, SF2, SF3 or others) as the response and the temperature (23°C or 27°C) and/or accession (Col-0 or Dobra-1) as effects and found to be not significant for either effects or their interaction (p>0.05).
(XLS)

S12 Table Primers used in this study. (XLS)
