# Supplementary figures and images for: Natural Variation Identifies ICARUS1, a Universal Gene Required for Cell Proliferation and Growth at High Temperatures in Arabidopsis thaliana
Source: PLoS Genet. 2015 May 7;11(5):e1005085. doi: 10.1371/journal.pgen.1005085 (PMC4423873; doi:10.1371/journal.pgen.1005085)

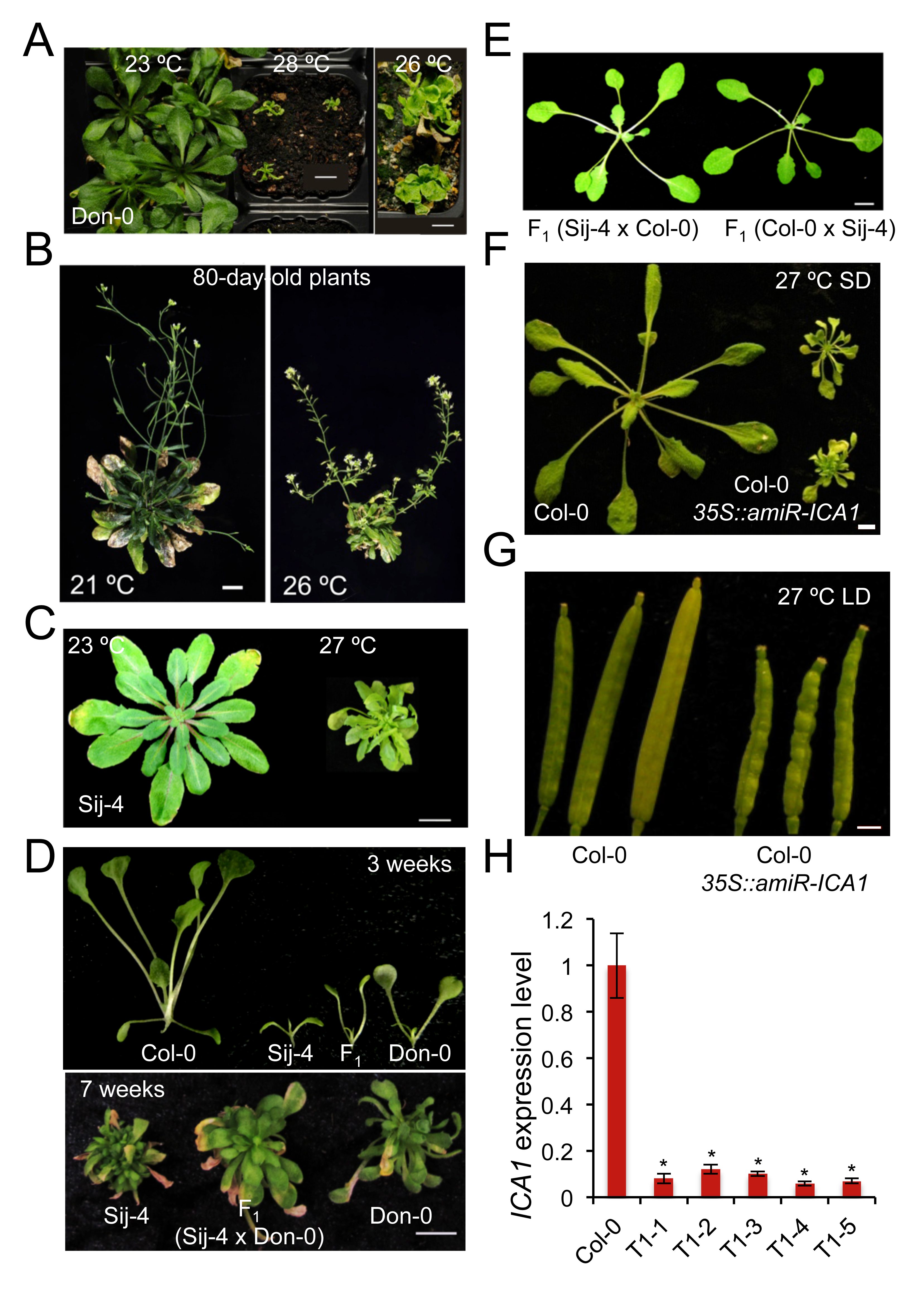

Supplement: S1 Fig — (A) 5-weeks-old Don-0 plants grown under long-day at 23°C or 28°C and 9-week-old plants grown at intermediate 26°C. (B) Don-0 plants at reproductive phase grown at 21 and 26°C. (C) 8-weeks-old Sij-4 plants grown under short-day at 23°C or 27°C. (D) ICA1-Sij-4 phenotype of F1 plants derived from crosses between Sij-4 and Don-0 grown at 27°C. (E) 4-weeks-old F1 (Sij-4 x Col-0) plants displaying normal development at 27°C. (F). 4-weeks-old 35S::amiR-ICA1 plants in Col-0 background compared with Col-0 at 27°C. (G) Malformed siliques produced by 35S::amiR-ICA1 plants grown under long-day (LD) conditions. (H) ICA1 expression level in 5 independent T1 35S::amiR-ICA1 transgenic lines in Col-0 background, normalized to tubulin. The expression levels are shown relative to that in Col-0. Error bars indicate ± standard errors based on technical replicates derived from three independent cDNAs. ***: p<0.0001. Scale bars: A, B & C = 12mm; D & F = 5mm; E = 6mm; G = 1mm. (TIF) [file pgen.1005085.s001.tif]

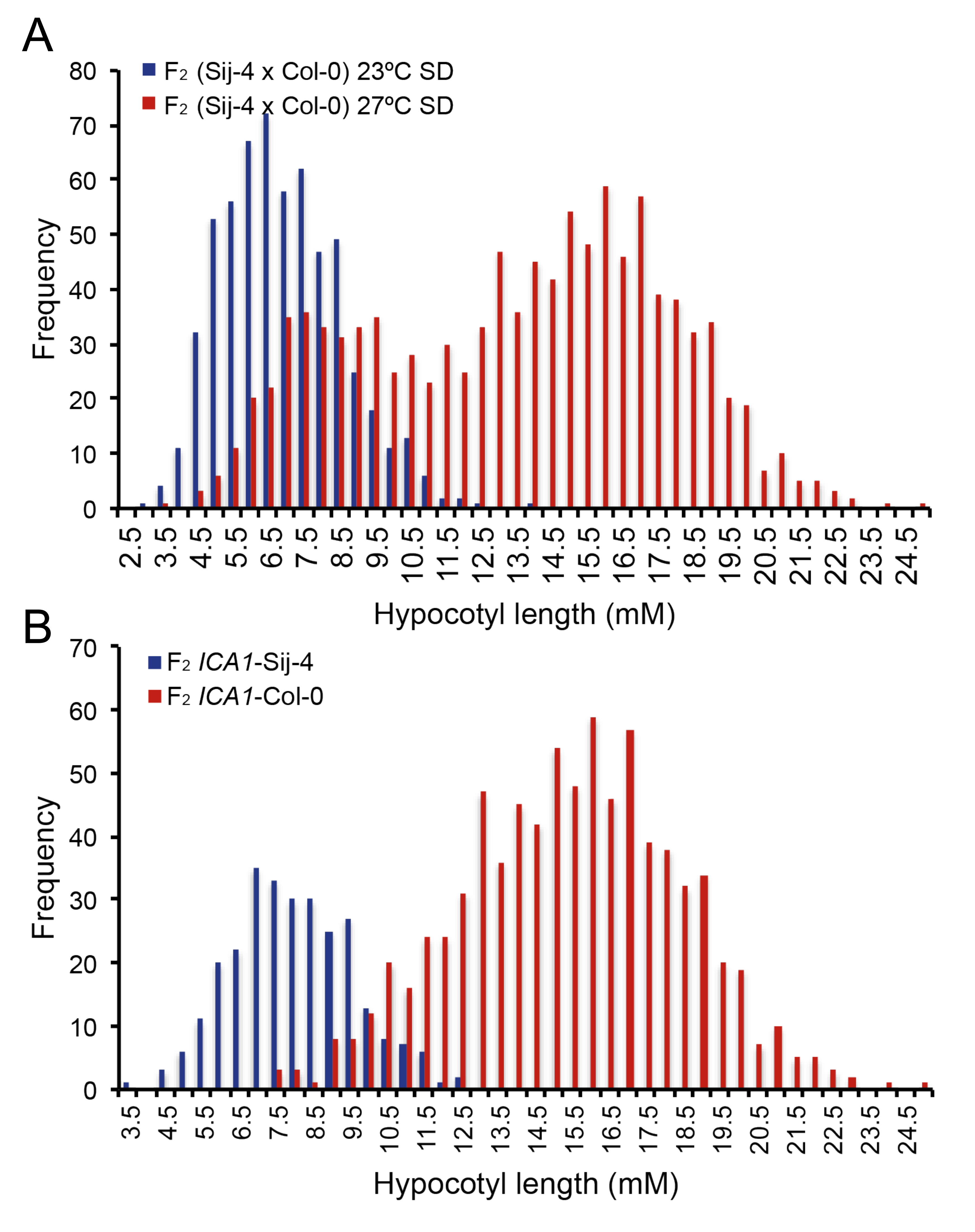

Supplement: S2 Fig — (A) Distribution of hypocotyl length in F2(Sij-4 x Col-0) plants grown at 23°C and 27°C. (B) Distribution of hypocotyl length in F2(Sij-4 x Col-0) grown at 27°C and classified according to ICA1 phenotype in leaves. The plants are color coded to differentiate the ICA1-Sij-4 plants (blue) and ICA1-Col-0 plants (red). Short hypocotyls co-segregate with leaf growth defect. (TIF) [file pgen.1005085.s002.tif]

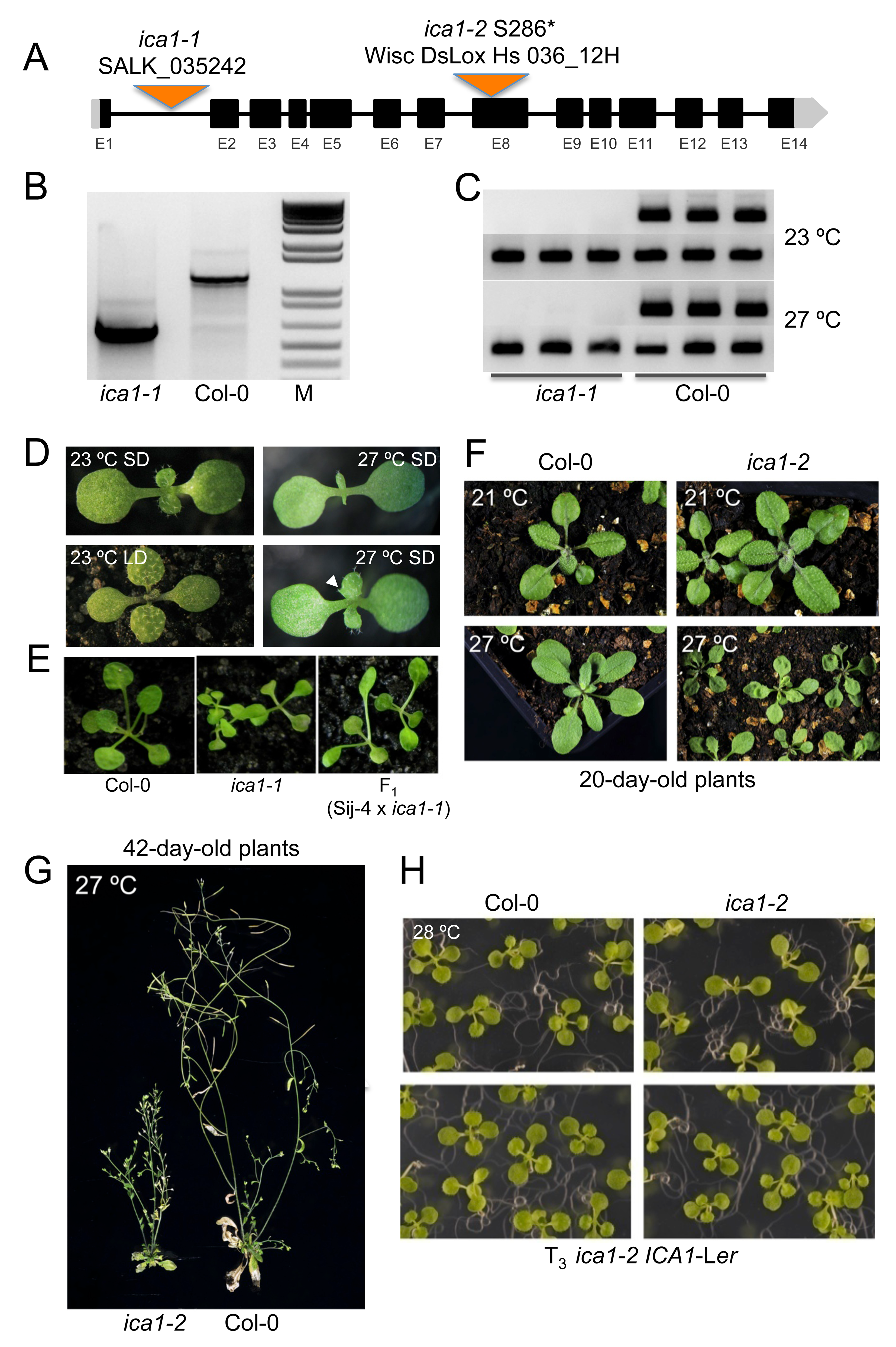

Supplement: S3 Fig — (A) Schematic representation of the T-DNA insertions at the ICA1 locus. (B) Confirmation of T-DNA insertion in ica1-1. Amplified products in Col-0 and ica1-1 plants with left border T-DNA primer along with primers flanking the insertion site. M:I kb+ ladder (C) Expression of ICA1 in 3 independent ica1-1 plants compared with Col-0. (D) Phenotypes of ica1-1 T-DNA line grown at different temperature and light conditions. The arrows indicate serrations in first leaves, which are not normally seen. Variable ica1 phenotype is identified in ica1-1 T-DNA lines indicating that this intronic insertion line is a partial loss-of-function allele. (E) The derived F1 plants between Sij-4 and ica1-1 insertion line grown at 27°C SD. (F) and (G) Vegetative and reproductive phase phenotypes of ica1-2 T-DNA line compared with Col-0 at 23°C or 27°C, respectively. (H) ica1-2 insertion line and its transgenic complementation by ICA1-Ler allele at 28°C. (TIF) [file pgen.1005085.s003.tif]

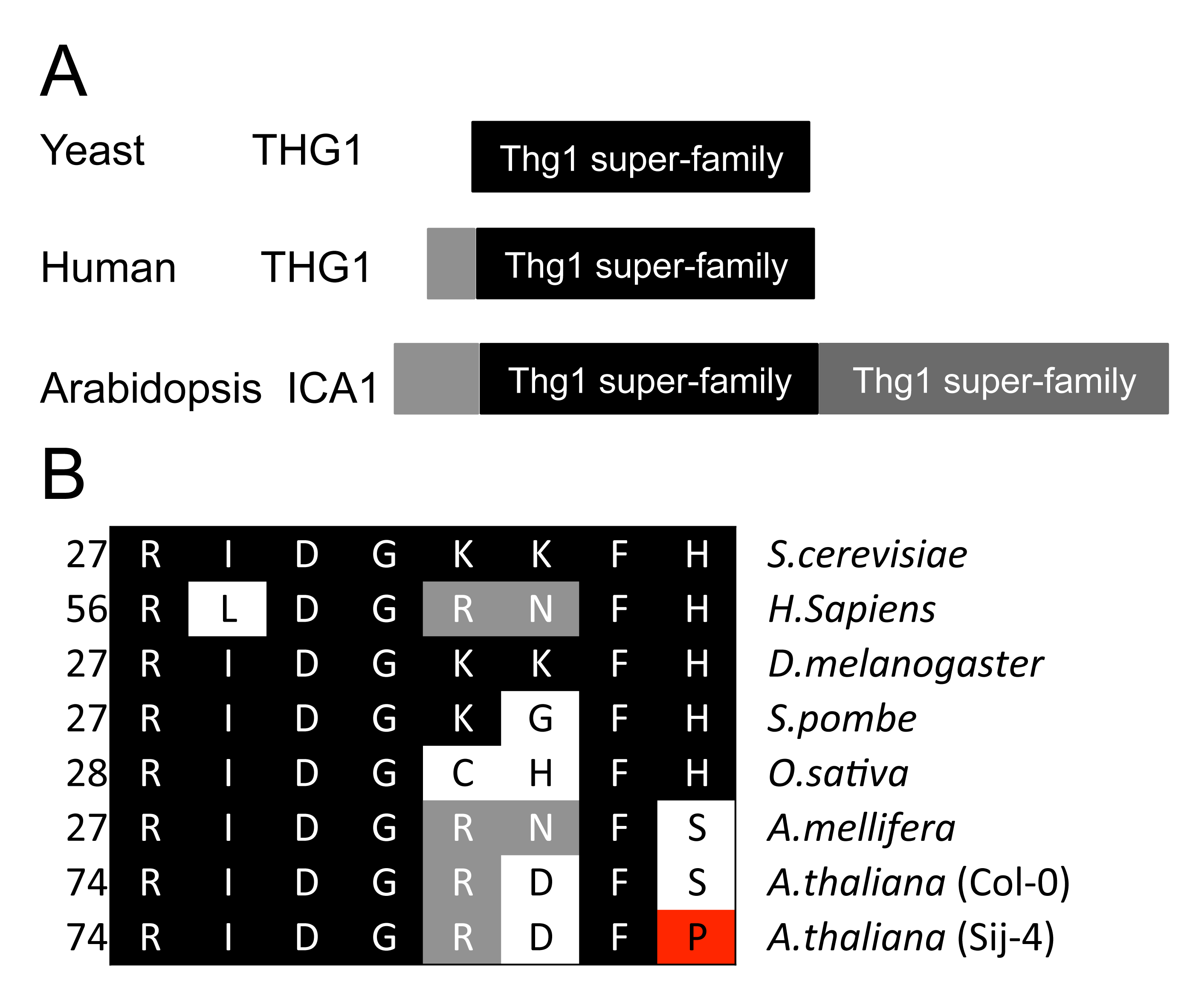

Supplement: S4 Fig — (A) Protein structure and homology between ICA1 from Arabidopsis and Thg1 from yeast and human. (B) Sequence alignment of the region containing S81P polymorphism in Sij-4 compared with Col-0 reference strain of Arabidopsis thaliana and the following species: Oryza sativa, Apis mellifera, Drosophila melanogaster, Saccharomyces pombe, Saccharomyces cerevisiae and Homo sapiens. Identical amino acids are shaded in black, while S81P, equivalent to yeast H34 and to the nucleotide binding site described in the human homologue [38] is marked in red color. (TIF) [file pgen.1005085.s004.tif]

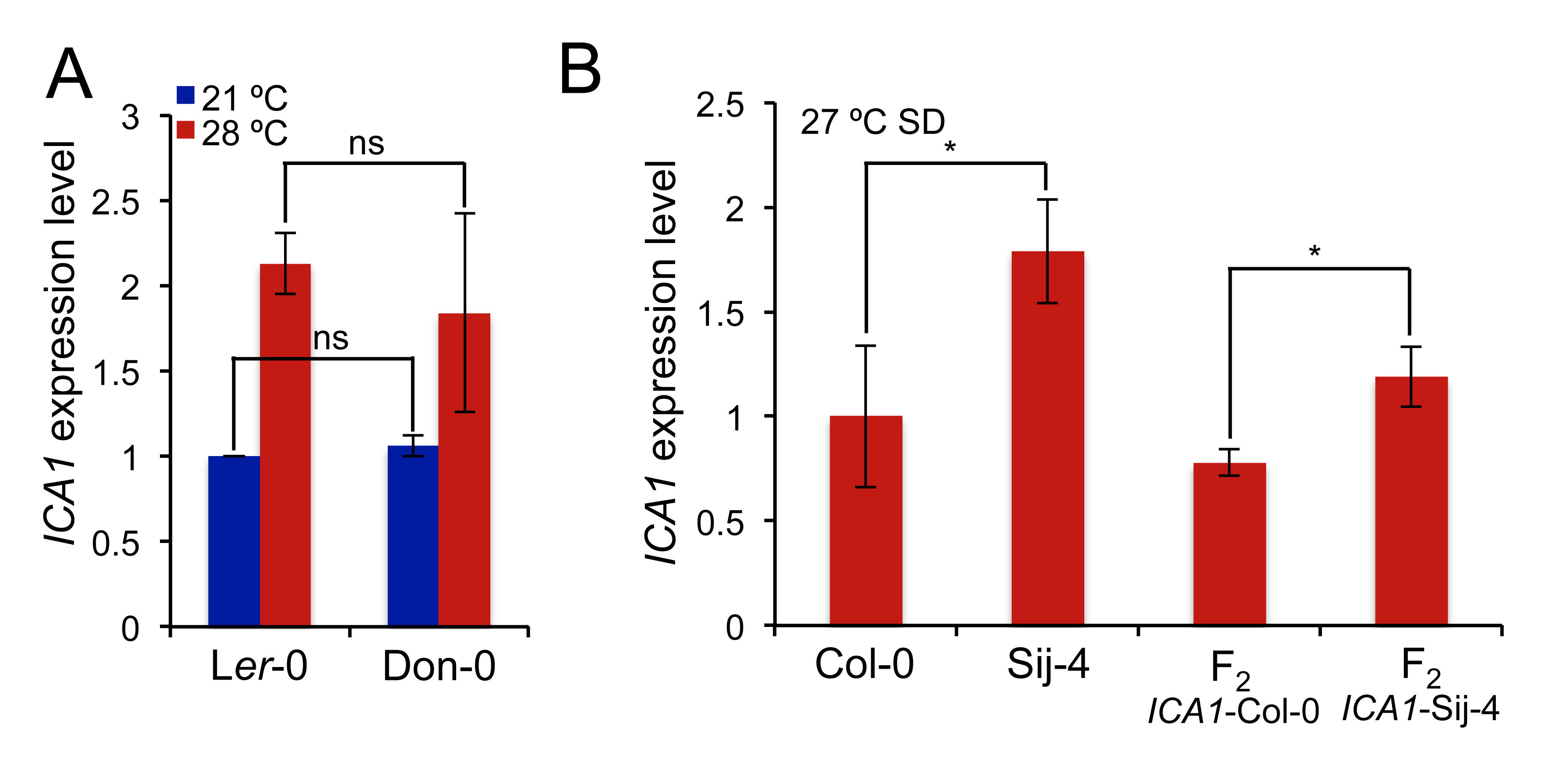

Supplement: S5 Fig — (A) Relative ICA1 expression levels in Ler and Don-0 grown at different temperatures under LD. (B) Relative ICA1 expression levels in Col-0 and Sij-4 accessions and in ICA1-Sij-4 and ICA1-Col plants selected from a F2 (Sij-4 x Col-0) grown under SD at 27°C, based on leaf phenotypes. Mean expression levels ± standard deviations are shown. ACTIN2 and TUB2/3 were used as internal controls for expression analyses in A and B, respectively. Ler and Don-0 did not differ statistically (ns: p>0.05), whereas differences between ICA1-Col and ICA1-Sij-4 were significant (*: p<0.05). (TIF) [file pgen.1005085.s005.tif]

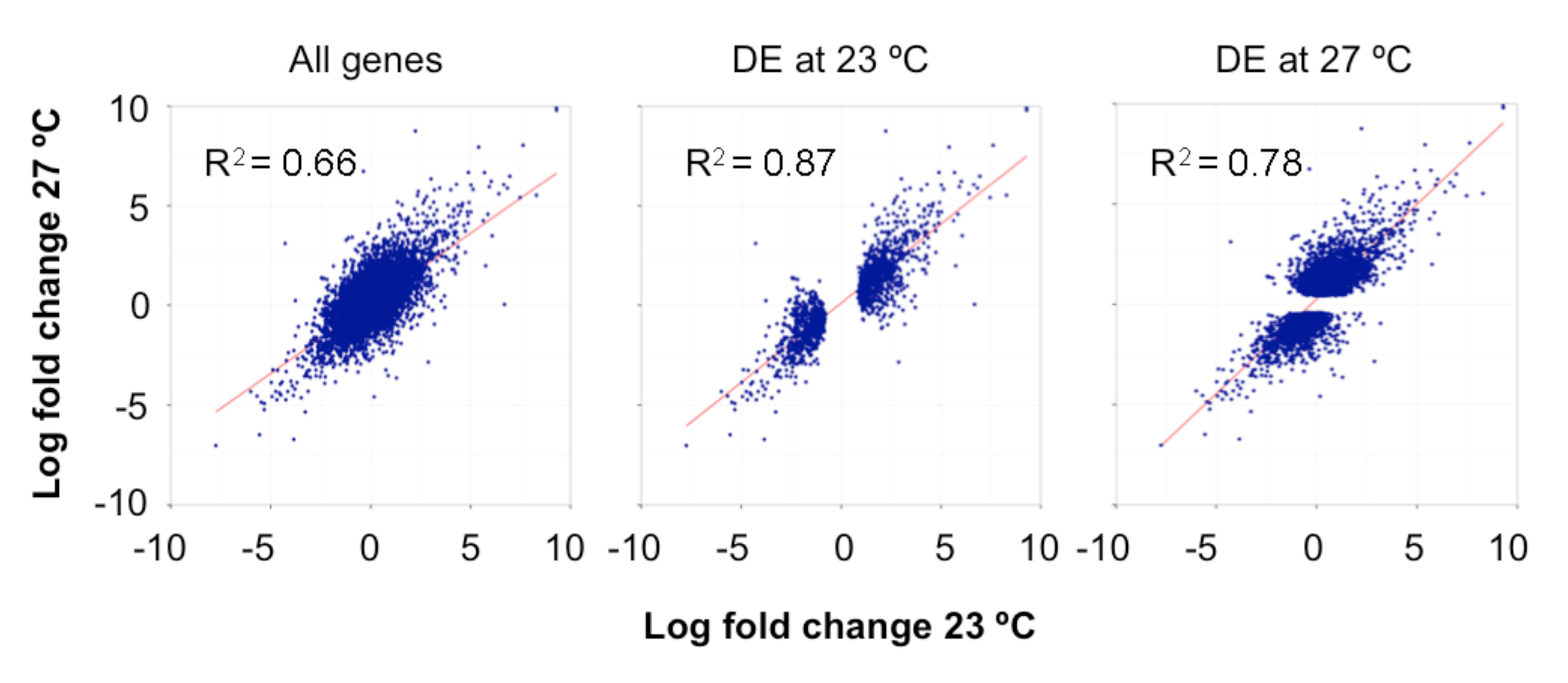

Supplement: S6 Fig — Log fold changes in gene expression between Col-0 and Sij-4 at 23°C and 27°C are plotted against each other and the R2 for the correlations are shown for all genes that were detected to be expressed (All genes) or for genes that are detected to be differentially expressed between Col-0 and Sij-4 at 23°C (DE at 23°C) or 27°C (DE at 27°C). Negative and positive log fold values indicate lower and higher Sij-4 expression in relation to Col. All correlations are significant (p<0.0001) suggesting that the directionality of changes in gene expression remain the same across temperatures and the differences are more pronounced at one or the other temperature. (TIF) [file pgen.1005085.s006.tif]

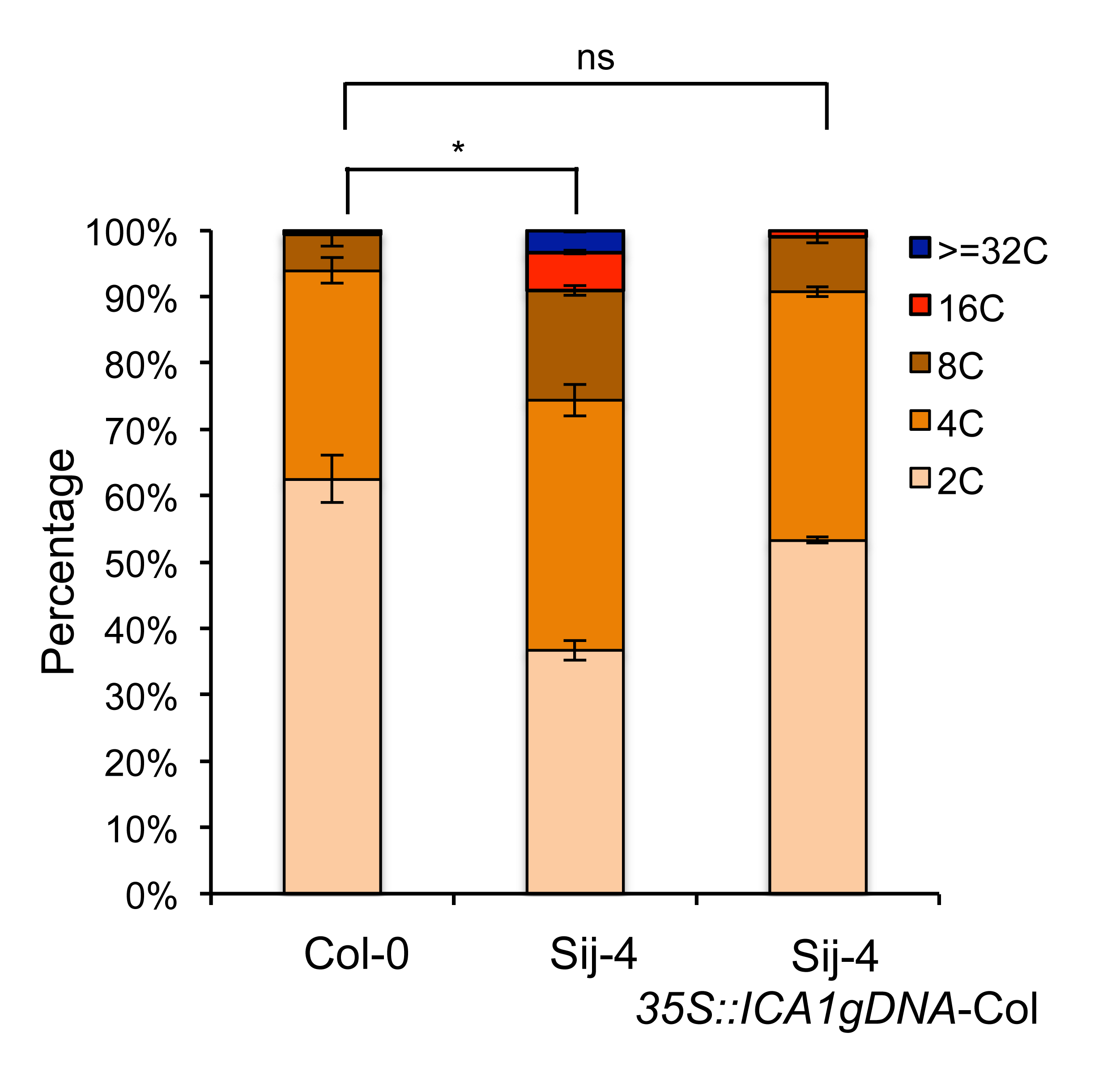

Supplement: S7 Fig — Proportion of nuclei with different DNA content in Col-0, Sij-4 and 35S::ICA1gDNA-Col in Sij-4 background. Mean values ± standard deviations are shown. The p-values obtained through Chi-square analysis are shown above. ns: p>0.05; *:p<0.0001. (TIF) [file pgen.1005085.s007.tif]

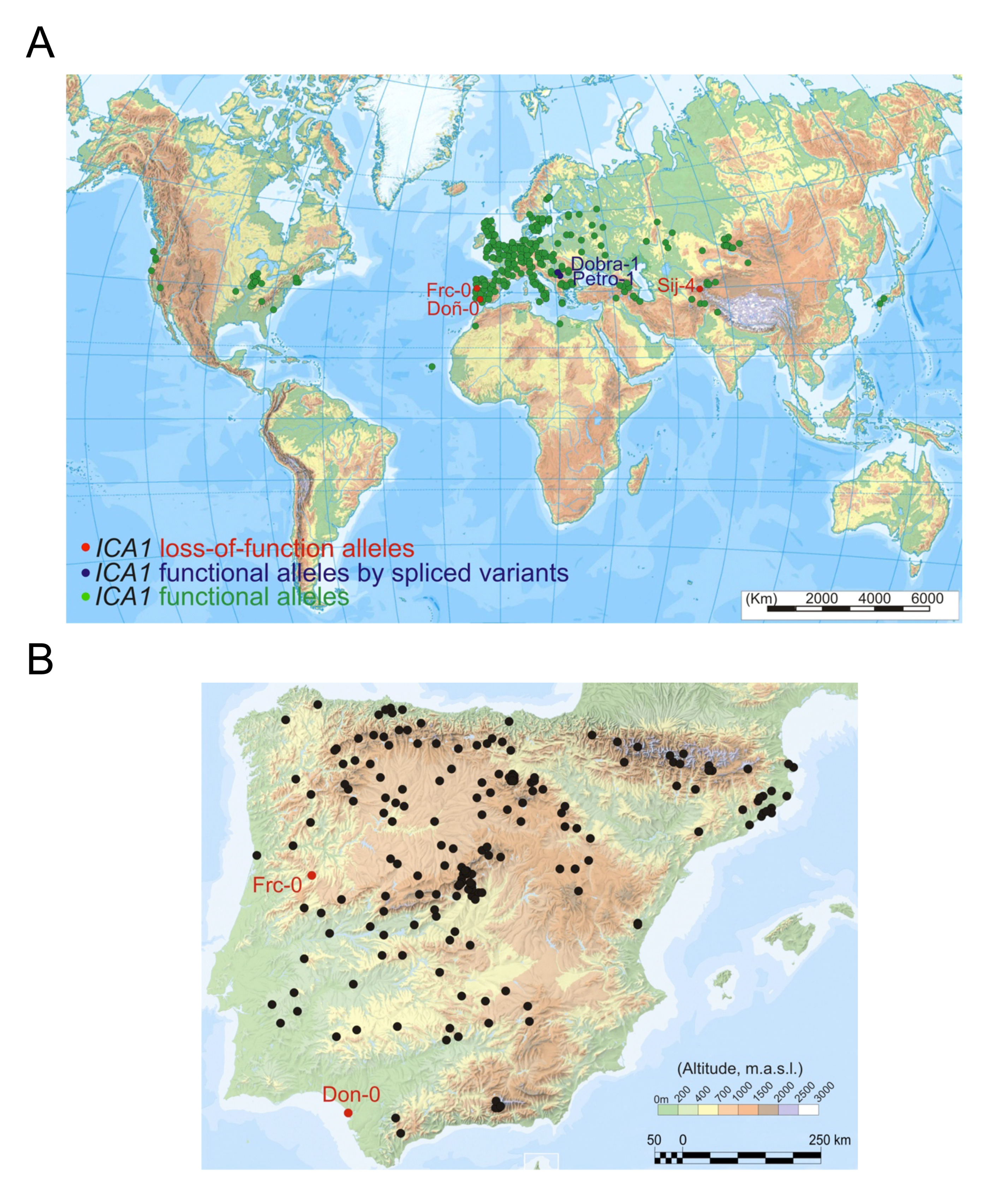

Supplement: S8 Fig — (A) Distribution of different ICA1 alleles across the globe. (B) Location of populations with high frequency of ICA1 loss-of-function alleles from the Iberian Peninsula. (TIF) [file pgen.1005085.s008.tif]

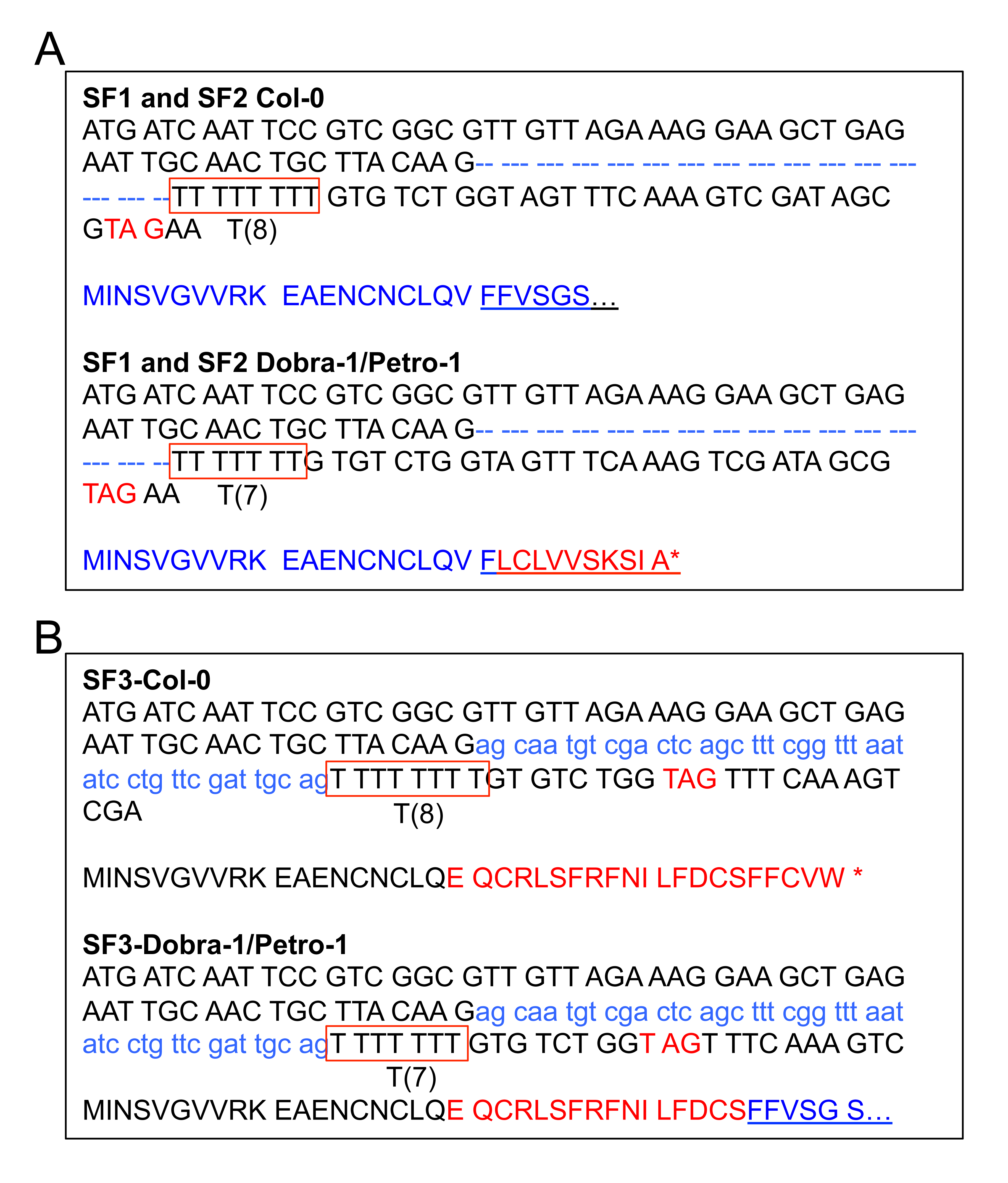

Supplement: S9 Fig — (A) Partial sequences of splice form 1 (SF1) and SF2 in Col-0 and Dobra-1/Petro-1 spanning the first intron and their predicted impacts on protein sequence. The protein sequence resulting from Dobra-1/Petro-1 allele, carrying a single bp (T) deletion that causes a frame shift, is shown in red. (B) Sequence of splice form 3 (SF3) in Col-0 showing the frame shift due to the partial intron retention and the corresponding protein sequence. The single bp (T) deletion of Dobra-1/Petro-1 restores the reading frame in SF3, which results in a protein similar to that encoded by SF1 with 15 additional amino acids. Intron sequences are shown in small blue letters and the additional amino acids are shown in red. (TIF) [file pgen.1005085.s009.tif]
